# Effect of Pathologic Tumor Response and Nodal Status on Survival in the Medical Research Council Adjuvant Gastric Infusional Chemotherapy Trial

**DOI:** 10.1200/JCO.2015.65.7692

**Published:** 2016-06-13

**Authors:** Elizabeth C. Smyth, Matteo Fassan, David Cunningham, William H. Allum, Alicia F.C. Okines, Andrea Lampis, Jens C. Hahne, Massimo Rugge, Clare Peckitt, Matthew Nankivell, Ruth Langley, Michele Ghidini, Chiara Braconi, Andrew Wotherspoon, Heike I. Grabsch, Nicola Valeri

**Affiliations:** Elizabeth C. Smyth, David Cunningham, William H. Allum, Alicia F.C. Okines, Clare Peckitt, Chiara Braconi, Andrew Wotherspoon, and Nicola Valeri, Royal Marsden Hospital; Andrea Lampis, Jens C. Hahne, Michele Ghidini, Chiara Braconi, and Nicola Valeri, The Institute of Cancer Research, London and Sutton; Matthew Nankivell and Ruth Langley, Medical Research Council Clinical Trials Unit at UCL, London; Heike I. Grabsch, University of Leeds, Leeds, United Kingdom; Matteo Fassan and Massimo Rugge, University of Padua, Padua, Italy; and Heike I. Grabsch, Maastricht University Medical Center, Maastricht, the Netherlands.

## Abstract

**Purpose:**

The Medical Research Council Adjuvant Gastric Infusional Chemotherapy (MAGIC) trial established perioperative epirubicin, cisplatin, and fluorouracil chemotherapy as a standard of care for patients with resectable esophagogastric cancer. However, identification of patients at risk for relapse remains challenging. We evaluated whether pathologic response and lymph node status after neoadjuvant chemotherapy are prognostic in patients treated in the MAGIC trial.

**Materials and Methods:**

Pathologic regression was assessed in resection specimens by two independent pathologists using the Mandard tumor regression grading system (TRG). Differences in overall survival (OS) according to TRG were assessed using the Kaplan-Meier method and compared using the log-rank test. Univariate and multivariate analyses using the Cox proportional hazards method established the relationships among TRG, clinical-pathologic variables, and OS.

**Results:**

Three hundred thirty resection specimens were analyzed. In chemotherapy-treated patients with a TRG of 1 or 2, median OS was not reached, whereas for patients with a TRG of 3, 4, or 5, median OS was 20.47 months. On univariate analysis, high TRG and lymph node metastases were negatively related to survival (Mandard TRG 3, 4, or 5: hazard ratio [HR], 1.94; 95% CI, 1.11 to 3.39; *P* = .0209; lymph node metastases: HR, 3.63; 95% CI, 1.88 to 7.0; *P* < .001). On multivariate analysis, only lymph node status was independently predictive of OS (HR, 3.36; 95% CI, 1.70 to 6.63; *P* < .001).

**Conclusion:**

Lymph node metastases and not pathologic response to chemotherapy was the only independent predictor of survival after chemotherapy plus resection in the MAGIC trial. Prospective evaluation of whether omitting postoperative chemotherapy and/or switching to a noncross-resistant regimen in patients with lymph node-positive disease whose tumor did not respond to preoperative epirubicin, cisplatin, and fluorouracil may be appropriate.

## INTRODUCTION

Patients with resectable gastric or gastroesophageal adenocarcinoma frequently undergo neoadjuvant therapy before surgical resection. The aim of this treatment is to down-stage primary tumors to achieve a microscopically complete resection, and to eliminate radiologically occult micrometastases, which may result in recurrent metastatic disease.^1-4^ Despite multimodal treatment, up to half of the patients who undergo surgery will subsequently relapse and die of their cancer. Identification of patients requiring additional therapy to prevent relapse remains challenging. No prognostic marker is currently available beyond standard pathologic Union for International Cancer Control TNM staging for patients with gastroesophageal cancer who receive neoadjuvant treatment.^5^^-8^

The UK Medical Research Council Adjuvant Gastric Infusional Chemotherapy (MAGIC) trial was an open-label, multicenter, phase III randomized trial comparing the effect of six cycles of perioperative epirubicin, cisplatin, and infused fluorouracil (ECF) chemotherapy plus surgery with surgery alone in patients with resectable gastroesophageal cancer.^2^ Patients treated with perioperative chemotherapy demonstrated improved overall survival (OS) compared with patients treated with surgery alone, and perioperative ECF chemotherapy is now recommended for selected patients by both the National Comprehensive Cancer Network and European Society for Medical Oncology guidelines.^9,10^ However, two key questions remain unanswered: whether we can define any biomarker that allows identification of patients at higher risk for recurrence after perioperative therapy and surgery and whether these high-risk patients might benefit from treatment with a different regimen. The latter question can only be answered by a randomized controlled trial; however, in an attempt to address the first question, herein we report the relationships among pathologic response, lymph node metastases, selected molecular abnormalities, neoadjuvant ECF chemotherapy, and survival in the MAGIC trial.

## MATERIALS AND METHODS

### Analysis of Pathologic Tumor Regression

Representative blocks with primary tumor or complete pathologic response were chosen by local pathologists and were collected centrally. Hematoxylin and eosin-stained slides were reviewed by two pathologists who were blinded to the treatment arm and graded for pathologic response according to the Mandard tumor regression grading (TRG) system.^11^ This system classifies pathologic response as follows: TRG 1 (complete regression/fibrosis with no evidence of tumor cells), TRG 2 (fibrosis with scattered tumor cells), TRG 3 (fibrosis and tumor cells with a dominance of fibrosis), TRG 4 (fibrosis and tumor cells with a dominance of tumor cells), and TRG 5 (tumor without evidence of regression). In cases of disagreement between pathologists, a consensus was sought by joint rereview and discussion. Histopathologic variables that had previously been collected were retrieved from the MAGIC database retained by the Medical Research Council Clinical Trials Unit at UCL, London, United Kingdom. The histologic tumor type according to Lauren’s classification^12^ was determined preferentially on the basis of pretreatment tumor biopsies and resection specimens if a biopsy was not available. The study was approved by the UK national ethical approval system before study commencement.

### Analysis of Tissue Biomarkers

Mutations in *KRAS*, *BRAF*, *PIK3CA, TP53*, and expression of phosphatase and tensin homolog (PTEN) and human epidermal growth factor receptor 2 (HER2) were assessed as previously described.^13-15^ The definition for HER2 positivity used was immunohistochemically 3+ or immunohistochemically 2+ and bright-field dual-probe in situ hybridization positive.

### Statistical Methods

OS was calculated from surgery to death from any cause or last date of follow-up.^2^ Date of surgery was selected as the baseline for biomarker analysis to avoid bias because patients treated with chemotherapy after being randomly assigned have a longer postrandomization survival. Date of surgery could not be confirmed for nine patients in the chemotherapy-plus-surgery arm, and these patients were excluded from the survival analyses. Differences in OS stratified by Mandard TRG were assessed using the Kaplan-Meier method and compared using the log-rank test. A *P* value of < .05 was considered significant.

χ^2^ tests were used to assess the effect of tissue biomarker status (*KRAS*, *BRAF*, *PIK3CA*, *TP53*, PTEN, and HER2) on pathologic response rate. The effect of tissue biomarker status on OS has previously been described.^13-15^

Univariate and multivariate analyses using the Cox proportional hazards method were performed to establish the relationships among age, sex, World Health Organization performance status (0 *v* 1), localization of the primary tumor, Lauren’s classification, TRG status, and presence of lymph node metastases on OS. Variables with a *P* value of < .05 in univariate analysis were included in the multivariate analysis.

## RESULTS

### Tumor Regression Grade, Lymph Node Status, and Patient Survival

**Fig 1.**
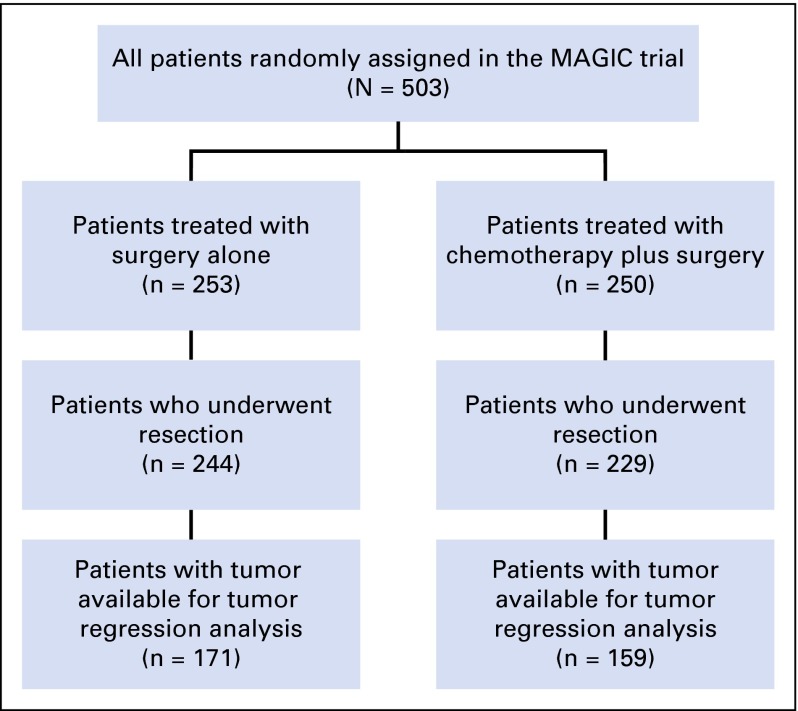
CONSORT diagram summarizing the analysis of pathologic tumor regression grading in the Medical Research Council Adjuvant Gastric Infusional Chemotherapy (MAGIC) trial. Tumor regression was assessed by two independent pathologists using the Mandard tumor regression grading system.

**Fig 2.**
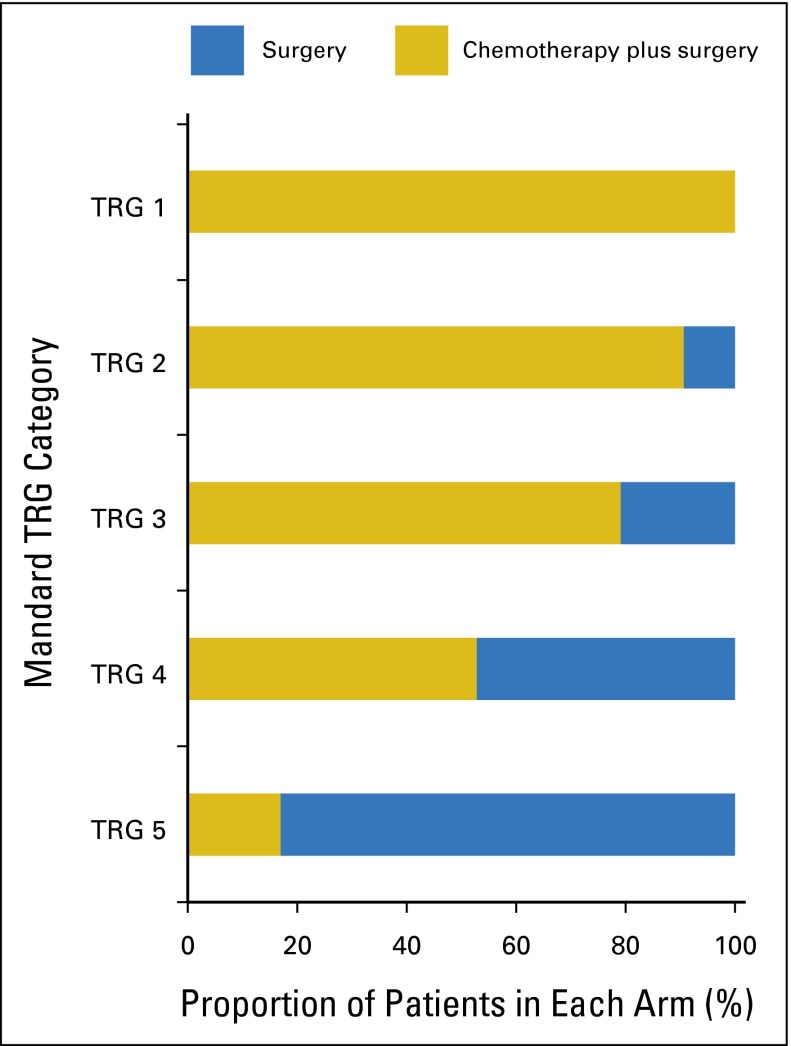
Tumor regression grade (TRG) and treatment in the Medical Research Council Adjuvant Gastric Infusional Chemotherapy (MAGIC) trial. Proportion of patients in each treatment arm according to TRG category. Tumors from patients treated with neoadjuvant chemotherapy were significantly more likely to show substantial tumor regression (TRG 1 or 2) than were tumors from patients treated with surgery alone (*P* < .001).

Five hundred three patients were randomly assigned in the MAGIC trial, 473 (94%) of whom underwent surgery. Three hundred thirty patients (171 from the surgery-alone arm, 159 from the chemotherapy-plus-surgery arm) had tissue available for tumor regression grading, representing 70% of patients who underwent surgery within the trial (Fig 1). Baseline characteristics of patients assessed for Mandard TRG are listed in Table 1. Consistent with the entire MAGIC trial population, most patients were male and most tumors were gastric cancers (76%) and intestinal type (80%). There was no significant difference in OS between patients treated with chemotherapy with and without tissue available for analysis (median OS, 23.1 months *v* 21.6 months, respectively; *P* = .264; Appendix Table A1, online only). The TRG results for patients treated with neoadjuvant chemotherapy were as follows: TRG 1 (n = 8, 5%); TRG 2 (n = 29, 18%); TRG 3 (n = 53, 34%); TRG 4 (n = 46, 29%); and TRG 5 (n = 23, 14%). Tumor regression-like changes were also seen in patients treated with surgery alone in the following proportions: TRG 1 (none); TRG 2 (n = 3, 2%); TRG 3 (n = 14, 8%); TRG 4 (n = 41, 24%); and TRG 5 (n = 113, 66%; Fig 2). The interobserver agreement between the two pathologists for TRG 1 to 5 was substantial, with a kappa of 0.64, which increased to 0.70 as expected when TRG was grouped as TRG 1 and 2 (responders) versus TRG 3 to 5 (nonresponders). Tumors from patients treated with neoadjuvant chemotherapy were significantly more likely to show significant tumor regression (TRG 1 or 2; *P* < .001 by Fisher’s exact test). Pathologic response to chemotherapy was not significantly associated with any clinicopathologic variable, including age, sex, site of tumor, or histologic subtype (Appendix Table A2, online only).

**Table 1.** Patient Characteristics

Characteristic	Surgery	Chemotherapy Plus Surgery
	No. (%)	No. (%)
Age category, years		
< 60	73 (42.7)	71 (44.7)
60 to < 70	59 (34.5)	52 (32.7)
≥ 70	39 (22.8)	36 (22.6)
Sex		
Female	46 (26.9)	31 (19.5)
Male	125 (73.1)	128 (80.5)
WHO score		
Normal activity	117 (68.4)	111 (69.8)
Restricted	54 (31.6)	48 (30.2)
Site		
Lower esophagus	22 (12.9)	22 (13.8)
Esophagogastric junction	19 (11.1)	16 (10.1)
Stomach	130 (76.0)	121 (76.1)
Histology		
Diffuse	34 (20.0)	24 (15.1)
Intestinal	124 (72.5)	130 (81.7)
Mixed, other	10 (5.8)	2 (1.3)
Not assessable	3 (1.7)	3 (1.9)

**Fig 3.**
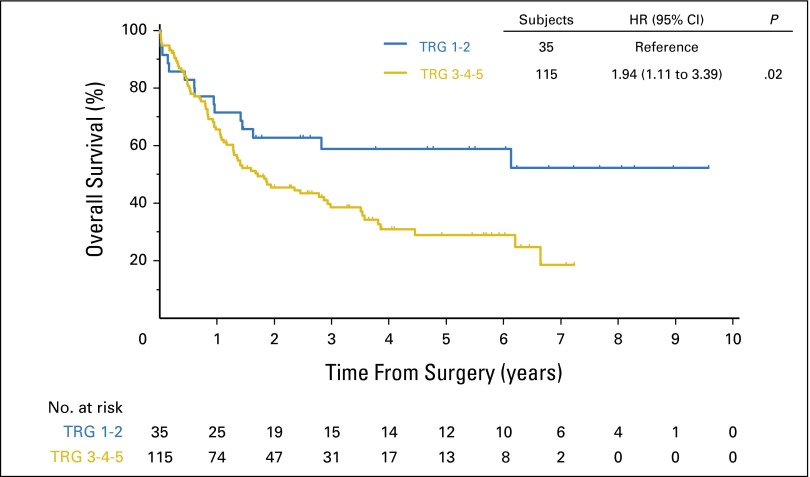
Overall survival by tumor regression grade (TRG) in patients treated with chemotherapy plus surgery in the Medical Research Council Adjuvant Gastric Infusional Chemotherapy (MAGIC) trial. Patients were dichotomized into two groups: TRG 1-2 responders and TRG 3-4-5 nonresponders. Differences in overall survival were assessed using the Kaplan-Meier method and compared using the log-rank test. A *P* value of < .05 was considered significant. HR, hazard ratio.

Because the survival of patients with TRG 1 and 2 was similar (data not shown) and the survival of patients with TRG 3, 4, or 5 also tracked together, the data set was dichotomized into two groups: TRG 1 or 2 (TRG 1-2) versus TRG 3, 4, or 5 (TRG 3-5) for further analyses (Table 2). Median OS for chemotherapy-treated patients with TRG 1-2 was not reached (lower limit of 95% CI, 17.28 months), whereas patients with a TRG of 3-5 had a median OS of 20.47 months (hazard ratio [HR], 1.94; 95% CI, 1.11 to 3.39; *P* = .0209; Fig 3). Five-year OS for chemotherapy-treated patients with TRG 1-2 was 58.8% (95% CI, 40.3% to 73.3%), whereas for chemotherapy-treated patients with TRG 3-5, it was 28.9% (95% CI, 19.5% to 38.9%), HR, 1.94 (95% CI, 1.11 to 3.39; *P* = .021).

**Table 2.** Overall Survival From Surgery Stratified by Mandard TRG in Patients Treated With Chemotherapy Plus Surgery

Mandard TRG	Median Survival	HR (95% CI)			P*
Mandard TRG (1 and 2 *v* 3 *v* 4 *v* 5)					
1-2	Not reached†				.098
3	22.51	1.86 (1.01 to 3.43)			
4	20.47	1.84 (0.97 to 3.49)			
5	19.15	2.43 (1.17 to 5.04)			
Mandard TRG (1 and 2 *v* 3 and 4 and 5)					
1-2	Not reached†	1.94 (1.11 to 3.39)			.0209
3-5	20.47				

Abbreviations: HR, hazard ratio; TRG, tumor regression grade.

* Cox regression method.

† Greater than last censoring time.

Details on lymph node dissection were available for 272 patients: in 138 patients (51%), fewer than 15 nodes were dissected; in 60 patients (22%), 15 to 20 lymph nodes were dissected; in 28 patients (10%), 21 to 25 lymph nodes were dissected; and in 46 patients (17%), more than 25 lymph nodes were dissected. The median number of lymph nodes removed was similar in the chemotherapy-plus-surgery arm (13; range, 0 to 63) and the surgery-alone arm (16; range, 0 to 91); this difference was not statistically significant (two-sample Mann-Whitney test *P* = .17).

Univariate Cox regression analysis including age, sex, performance status, site of primary tumor, lymph node status, and TRG demonstrated that both TRG (1-2 *v* 3-5) and lymph node status (node-negative [ypN0] *v* node-positive [ypN+]) were significantly associated with OS in chemotherapy-treated patients (TRG 3-5: HR, 1.94; 95% CI, 1.11 to 3.39; *P* = .0209; ypN+: HR, 3.63; 95% CI, 1.88 to 7.0; *P* = .0001; Appendix Table A3, online only). Multivariate analysis including TRG and lymph node status performed in 110 patients for whom all clinical-pathologic information were available demonstrated that the presence of lymph node metastases was the only factor independently predictive of OS in patients after neoadjuvant chemotherapy (HR, 3.36; 95% CI, 1.70 to 6.63; *P* < .001; Appendix Table A4, online only).

**Fig 4.**
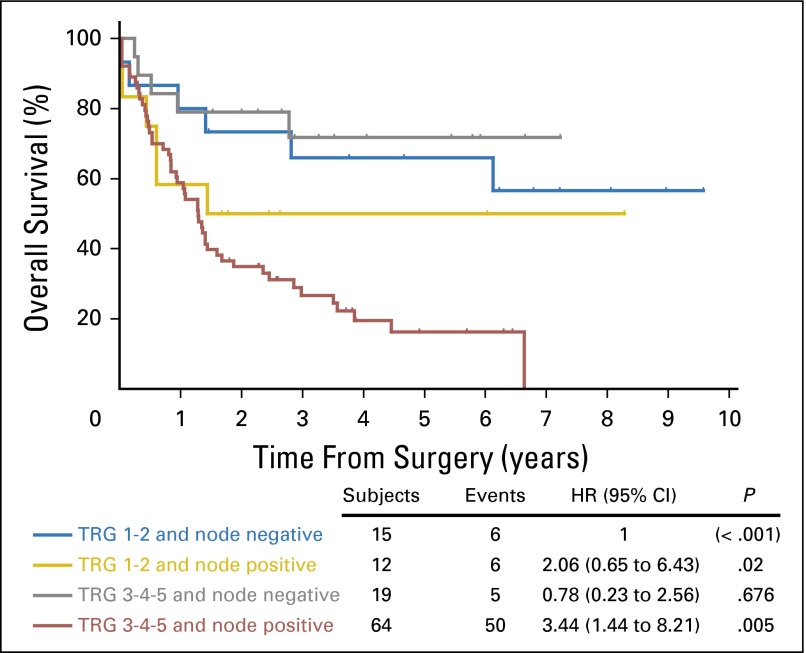
Overall survival by tumor regression grade (TRG) and lymph node status in patients treated with chemotherapy plus surgery in the Medical Research Council Adjuvant Gastric Infusional Chemotherapy (MAGIC) trial. Patients were stratified into four groups: ypN0 and TRG 1 or 2 (node-negative responders); ypN1+ TRG 1 or 2 (node-positive responders); ypN0 and TRG 3, 4, or 5 (node-negative nonresponders); and ypN1+ and TRG 3, 4, or 5 (node-positive nonresponders). Differences in overall survival were assessed using the Kaplan-Meier method and compared using the log-rank test. A *P* value of < .05 was considered significant. HR, hazard ratio.

A statistical model was created containing four groups of chemotherapy-treated patients; (A) ypN0 and TRG 1 or 2 (node-negative responders); (B) ypN1+ and TRG 1 or 2 (node-positive responders); (C) ypN0 and TRG 3, 4, or 5 (node-negative nonresponders); and (D) ypN1+ and TRG 3, 4, or 5 (node-positive nonresponders). The median OS for all node-negative patients (groups A and C, regardless of TRG status) was not reached because it was greater than the longest censoring time, whereas the median OS for node-positive responders (group B) was 17.3 months (95% CI, 0.5 to not reached) and that for node-positive nonresponders (group D) was 15.5 months (95% CI, 10.2 to 19.2 months); these differences were statistically significant (*P* < .001; Fig 4). The 5-year OS rates for groups A, B, C, and D were 66.0% (95% CI, 36.5% to 84.3%), 50.0% (95% CI, 20.9% to 73.6%), 71.8% (95% CI, 44.3% to 87.4%), and 16.2% (95% CI, 7.2% to 28.4%), respectively (*P* = .001). In comparison, median OS in the surgery-alone arm for node-positive patients (with TRG results available) was 19.0 months (95% CI, 14.1 to 25.0 months).

### Correlation of TRG and Lymph Node Status With Molecular Biomarker Status

Mutations in *KRAS* (codons 12 and 13), *BRAF*, *PIK3CA* (exons 9 and 20), and *TP53* (exons 4-8) were present in 6.4%, 0.7%, 5%, and 37.9% of patients treated with chemotherapy who had TRG status available, respectively. TRG 1-2 response was not documented in any patient with a *KRAS*, *BRAF*, or *PIK3CA* mutation; however, none of these genes was individually statistically significantly associated with pathologic response to chemotherapy. When all *KRAS*, *BRAF*, and *PIK3CA* mutations (n = 16) were combined as an oncogene mutant group, the association with lack of response reached borderline statistical significance (*P* = .055). Fourteen of 36 patients (38%) with a *TP53* mutation had a TRG 1 or 2 pathologic response compared with 14 of 59 patients (24%) who did not; this difference was not statistically significant (*P* = .116). In patients with low or no PTEN expression (38% of the cohort) with TRG results, 12 of 56 (21%) had TRG 1-2, compared with 15 of 92 patients (16%) with intact PTEN. One of 13 patients (7.7%) who were HER2 positive with a TRG result had a TRG of 1-2, compared with 31 of 141 (21.9%) who were HER2 negative (*P* = .224). There was no statistically significant association between *KRAS*, *BRAF*, *PIK3CA*, *TP53*, PTEN, or HER2 status and the presence of involved lymph nodes in the resection specimen.

## DISCUSSION

To our knowledge, this study is the first to report the results of interaction between the prognostic effects of centrally analyzed tumor regression and other clinicopathologic variables on survival in a randomized trial with a nonchemotherapy control arm for perioperatively treated gastroesophageal cancer. We demonstrated on univariate analysis that tumor regression (TRG 1 or 2, eg, complete pathologic response or low number of residual tumor cells) is associated with improved OS in chemotherapy-treated patients, but not in surgery-alone–treated patients. However, the relationship between TRG and survival is not independent of lymph node status, which was the only independent predictor of survival in patients treated with chemotherapy in this study.

A strength of our study is that all available hematoxylin and eosin slides from the resection specimens were subjected to central pathology review, which guaranteed relatively uniform classification of tumor regression according to Mandard.^11,12^ Previously published studies in this field were all retrospective, were usually performed with single-center nonrandomized cohorts, used a variety of different tumor regression grading systems, and lacked a surgery-alone control group. Our results validate the results of the largest previous uncontrolled series, which also reported that lymph node status is more important as a prognostic variable than tumor regression.^16^ This finding underscores the paramount importance of adequate lymph node resection for accurate staging and, hence, prognosis prediction in patients with gastroesophageal cancer.^16-18^ Because there is no independent effect of pathologic tumor response to chemotherapy, we hypothesize that the survival benefit demonstrated in the chemotherapy-plus-surgery arm in the MAGIC trial may be due to tumor down-staging (and more R0 resections) because lower T stages and lower rates of lymph node involvement were seen in the chemotherapy arm of the MAGIC trial; however, this could also be a result of the effect of chemotherapy on micrometastatic disease, which is not measured.

We also examined the effect of several molecular abnormalities on lymph node status and response to chemotherapy. Interestingly, no significant pathologic responses to chemotherapy were detected in any patient with a *KRAS*, *BRAF*, or *PIK3CA* mutation. The chemoresistant effect of *RAS* or *PIK3CA* pathway activation has been described in non-small cell lung cancer and colorectal preclinical models and clinically in patients with cervical cancer; therefore, this may be a true effect. However, the current analysis is limited by the small proportion of patients with mutations.^19-21^ Patients who were HER2 positive also appeared less likely to demonstrate a significant pathologic response to ECF chemotherapy. Preclinical data to support this result have recently been described; however, HER2-positive patients with esophagogastric cancer have not previously been demonstrated to have inferior response rates to chemotherapy.^22^ In the Trastuzumab for Gastric Cancer trial, the radiologic response rate to cisplatin-fluoropyrimidine therapy was 35%, which is consistent with other similar data sets.^23,24^ We have also previously demonstrated that HER2 status in MAGIC was not associated with differential survival outcomes, and we believe that this result requires further verification.^13^

As Lauren’s diffuse tumors may have a significant stromal element, it is challenging to perform pathologic response assessment in this subtype of gastric cancer. In our study, neither Lauren’s histologic subtype was statistically significantly more likely to demonstrate a good pathologic response to chemotherapy overall. However, we did not evaluate the presence of signet ring cells, which have in several series been associated with reduced rates of response to chemotherapy.^25^ It is possible that this unique group of patients may have different outcomes; however, because only 18% of patients (n = 58) in our cohort had Lauren’s diffuse cancer, the current analysis may be underpowered to evaluate this subset.^26^

A potential limitation of our analysis is that we were unable to include all patients from the MAGIC trial in this study because we did not receive material from the entire patient cohort. However, because survival after chemotherapy was not different for those who did not have tissue available for analysis compared with the patients analyzed, we do not believe that this introduced significant bias. The optimal system for assessment of pathologic tumor regression is contentious.^27,28^ We chose the Mandard system, and although we acknowledge that this classification system was initially designed for patients with esophageal squamous cell cancer undergoing chemoradiotherapy, it is the most widely used system in esophagogastric cancer. Alternative systems include those proposed by Becker et al,^29^ Dworak et al,^30^ and Rödel et al.^31^ The Dworak et al^30^ and Rödel et al^31^ systems were designed for rectal adenocarcinomas following chemoradiotherapy, and the Becker et al^29^ system (which was designed specifically for assessment in chemotherapy-treated patients with gastric cancer) requires review of the entire tumor bed, which was not available for all MAGIC specimens. Because only a representative block was chosen for this assessment, it is therefore likely that the block with the most tumor was chosen, and if the underlying cancer demonstrates heterogeneity of response to chemotherapy, then the assessment will be biased toward nonresponders. Reassuringly, our findings are similar to those from a large study published using the Becker et al^29^ criteria, in which tumor stage (incorporating lymph node status) but not tumor regression was an independent prognostic factor after neoadjuvant therapy.^16^

In the MAGIC trial, fewer than half of patients completed all protocol chemotherapy. Because postoperative morbidity after esophagogastrectomy is considerable, there is often a sound clinical rationale for this. For these patients, the absence of a significant pathologic response in the resection specimen may lead to reluctance to complete postoperative chemotherapy. These data are not presented with the intention of influencing any change in practice with respect to use of perioperative chemotherapy; however, it is intuitively tempting to directly compare the median survival of node-positive nonresponders in the chemotherapy arm with node-positive surgery alone. Chemotherapy-treated patients who are node positive after surgery and who have no significant pathologic response (TRG 3, 4, or 5) in the resected primary tumor have a survival outcome inferior to those who were not treated with chemotherapy. However, it is possible that the survival of those in the chemotherapy arm could have been even worse without chemotherapy. Finally, for the avoidance of nihilism, it is also important to note that even the patients with a poor pathologic response have a chance of cure (28.9% 5-year survival in our model). Thus, even a modest response to chemotherapy may play an important role in survival outcomes, and TRG may not be sensitive to these changes. The only firm conclusion that can currently be made is that node-positive nonresponders are a relatively poor prognosis group, and only a future randomized trial can accurately determine whether changing or intensifying treatment of nonresponders will result in improvements in OS for these patients.

In conclusion, our study demonstrates that in patients with gastroesophageal cancer treated with perioperative ECF chemotherapy, the lymph node status in the resection specimen and not the regression of the primary tumor is the primary arbiter of survival. Although ^18^F-labeled fluorodeoxyglucose-positron emission tomography/computed tomography response in the primary tumor has been validated as a predictive marker of OS after a single cycle of chemotherapy in two studies, we do not know how this relates to lymph nodes status nor whether switching to a noncross-resistant chemotherapy regimen will result in improved survival^32-34^; this question may be answered by an ongoing US clinical trial (NCT02485834), in which patients who are ^18^F-labeled fluorodeoxyglucose-positron emission tomography nonresponders are randomly assigned to surgery followed by chemoradiation or to salvage perioperative chemotherapy. In the interim, because the median survival for patients with resectable Western gastroesophageal cancer undergoing potentially curative surgery is fewer than 3 years, further work is required to identify more effective therapies and improve outcomes.^8^

## Supplementary Material

Protocol

## Appendix

**Table A1.** Comparison of OS (calculated from time of surgery) in Patients in the Chemotherapy-Plus-Surgery Arm With and Without Mandard TRG Score

	Subjects	Events	Median OS	95% CI	*P*
TRG available	150	90	23.4	16.8 to 42.4	
TRG not available	65	39	21.6	9.6 to NR	.645

Abbreviations: NR, not reached; OS, overall survival; TRG, tumor regression grade.

**Table A2.** Association Between Clinicopathologic Variables and Response to Chemotherapy

Variable	Odds Ratio (95% CI)	*P*
Age, years		
Overall		.744
< 60		
60 to 70	1.37 (0.60 to 3.18)	.455
≥ 70	1.07 (0.40 to 2.82)	.896
Site		
Overall		.14
Lower esophagus		
Esophagogastric junction	6.00 (1.02 to 35.3)	.047
Stomach	3.16 (0.69 to 14.3)	.137
Sex		
Female		
Male	0.84 (0.34 to 2.08)	.709
Histology		
Diffuse		
Others	0.51 (0.20 to 1.31)	.163

**Table A3.** Univariate Analysis of Factors Affecting Overall Survival in Patients Treated With Chemotherapy Plus Surgery (n = 150)

Variables	HR (95% CI)	*P*
Age, years		
< 60		.122
60 to < 70	1.42 (0.88 to 2.30)	.150
≥ 70	1.70 (1.00 to 2.90)	.051
Sex		
Female		
Male	1.60 (0.89 to 2.88)	.117
WHO score		
Normal activity		
Restricted	1.10 (0.71 to 1.72)	.669
Site		
Lower esophagus		.113
Esophagogastric junction	0.39 (0.15 to 1.01)	.052
Stomach	0.63 (0.36 to 1.10)	.106
Histology		
Diffuse	.754	
Intestinal	1.11 (0.62 to 2.00)	.702
MI + diff/other	1.10 (0.14 to 8.41)	.928
Not assessed	3.12 (0.40 to 24.2)	.276
TRG score		
1-2		
3-5	1.94 (1.11 to 3.39)	.021
N stage		
Node-negative		
Node-positive	3.63 (1.88 to 7.00)	< .001

Abbreviation: HR, hazard ratio; MI + diff/other, mixed intestinal and diffuse or other; TRG, tumor regression grade.

**Table A4.** Multivariate Analysis of Factors Affecting Overall Survival in Patients Treated With Chemotherapy Plus Surgery (n = 110)

Variable	HR (95% CI)	*P*
TRG		
3-5	1.32 (0.69 to 2.52)	.411
	
N stage		
Node positive	3.36 (1.70 to 6.63)	< .001

Abbreviations: HR, hazard ratio; TRG, tumor regression grade.
