# Supplementary material for: Effect of Pathologic Tumor Response and Nodal Status on Survival in the Medical Research Council Adjuvant Gastric Infusional Chemotherapy Trial
Source: J Clin Oncol. 2016 Jun 13;34(23):2721–7. doi: 10.1200/JCO.2015.65.7692 (PMC5019747; doi:10.1200/JCO.2015.65.7692)
Supplement: Protocol [file supp_34_23_2721__index.html]

Effect of Pathologic Tumor Response and Nodal Status on Survival in the Medical Research Council Adjuvant Gastric Infusional Chemotherapy Trial — Tumor Regression Grading and Survival in MRC MAGIC Trial — Protocol 

# Effect of Pathologic Tumor Response and Nodal Status on Survival in the Medical Research Council Adjuvant Gastric Infusional Chemotherapy Trial

## Protocol

The following protocol information is provided solely to describe how the authors conducted the research underlying this article. The information provided may not reflect the complete protocol or any previous amendments or modifications. As described in the Information for Contributors (http://jco.ascopubs.org/site/ifc/protocol.xhtml), *JCO* requests only specific elements of the most recent version of the protocol. The protocol information is not intended to replace good clinical judgment in selecting appropriate therapy and in determining drug doses, schedules, and dose modifications. The treating physician or other health care provider is responsible for determining the best treatment for the patient. ASCO and *JCO* assume no responsibility for any injury or damage to persons or property arising out of the use of this protocol material or due to any errors or omissions. Readers seeking additional information about the protocol are encouraged to consult the corresponding author directly.

Please click the protocol link below to access the information.

**Files in this Data Supplement:**

- Protocol
