# Supplementary material for: Effect of Pathologic Tumor Response and Nodal Status on Survival in the Medical Research Council Adjuvant Gastric Infusional Chemotherapy Trial
Source: J Clin Oncol. 2016 Jun 13;34(23):2721–7. doi: 10.1200/JCO.2015.65.7692 (PMC5019747; doi:10.1200/JCO.2015.65.7692)
Supplement: Protocol [file supp_JCO.2015.65.7692_Protocol_2015.657692.docx]

The following protocol information is provided solely to describe how the authors conducted the research underlying the published report associated with the following article:

**Effect of pathological tumour response and nodal status on survival in the Medical Research Council Adjuvant Gastric Infusional Chemotherapy (MAGIC) TrialFirst**

**Smyth, et al**

DOI: 10.1200/JCO.2015.65.7692

The information provided may not reflect the complete protocol or any previous amendments or modifications. As described in the Author Center (<http://jco.ascopubs.org/site/ifc/manuscript-guidelines.xhtml#randomized_phase_one_and_two>) only specific elements of the most recent version of the protocol are requested by JCO. The protocol information is not intended to replace good clinical judgment in selecting appropriate therapy and in determining drug doses, schedules, and dose modifications. The treating physician or other health care provider is responsible for determining the best treatment for the patient. ASCO and JCO assume no responsibility for any injury or damage to persons or property arising out of the use of these protocol materials or due to any errors or omissions. Individuals seeking additional information about the protocol are encouraged to consult with the corresponding author directly.


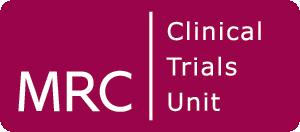


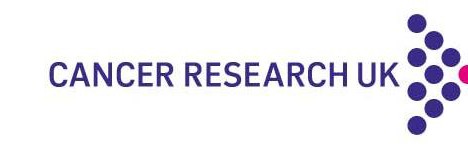


**TransMAGIC**

Translational Study for the MRC Adjuvant Gastric Cancer Infusional Chemotherapy Trial.

TransMAGIC Translational Research Protocol

**Version 2.0: 2nd March 2015**

**MAGIC trial REC reference number:** 97/1/66

**ISRCTN:** 9379397

## RMH CCR Number: 1043

| **Authorised by:** | | | |
| --- | --- | --- | --- |
| Name: | **Professor David Cunningham** | Role: | **Chief Investigator** |
| Signature: |  | Date: |  |

### Trial Administration:

Please direct all queries to the TransMAGIC Clinical Coordinator, Dr Elizabeth Smyth at the Royal Marsden Hospital.

**nator**

**myth**

rsden

| **Chief Investigator**  **Professor David Cunningham** The Royal Marsden  Downs Road Sutton Surrey  SM2 5PT | |  |  |
| --- | --- | --- | --- |
| Tel:  Fax: Email: | +44 (0) 208 661 3156  +44 (0) 208 643 9414  [David.Cunningham@rmh.nhs.uk](mailto:David.Cunningham@rmh.nhs.uk) | Tel: +44 (0) 208 642 6011  Fax: +44 (0) 208 661 3890  Email: Elizabeth [.smyth@rmh.nhs.uk](mailto:.smyth@rmh.nhs.uk) | |

**Stenning**

L

|  | |  |  |
| --- | --- | --- | --- |
| **Sponsor Representative**  **Michelle Gabriel** Cancer Group MRC CTU at UCL  Aviation House 125 Kingsway London  WC2B 6NH  United Kingdom | |  |  |
| Tel: Fax: Email: | +44 (0) 20 7670 4700  +44 (0)20 7670 4818  [m.gabriel@ucl.ac.uk](mailto:m.gabriel@ucl.ac.uk) | Tel: +44 (0) 20 7670 4700  Fax: +44 (0)20 7670 4818  Email: [s.stenning@ucl.ac.uk](mailto:s.stenning@ucl.ac.uk) | |

### Senior Research Scientist/ Oncologist

**Dr Ruth Langley** Cancer Group MRC CTU at UCL

Aviation House 125 Kingsway London

WC2B 6NH

### Consultant Histopathologist

**Dr Andrew Wotherspoon** The Royal Marsden

Department of Histopathology Downs Road

Sutton Surrey SM2 5PT

Tel:

Fax: Email:

+44 44 (0) 20 7670 4700

+44 (0) 207 670 4685

[r.langley@ucl.ac.uk](mailto:r.langley@ucl.ac.uk)

Tel:

Fax: Email:

+44 (0) 207 352 8171 x2560

[Andrew.Wotherspoon@rmh.nhs.uk](mailto:Andrew.Wotherspoon@rmh.nhs.uk)

### Royal Marsden Statistician

Clare Peckitt

The Royal Marsden Downs Road Sutton

Surrey SM2 5PT

Royal Marsden Clinician Scientist

Dr Nicola Valeri

Centre for Molecular Pathology The Royal Marsden

Downs Road Sutton Surrey

Tel: Email:

+44 (0) 207 352 8171 x6269

[clare.peckitt@rmh.nhs.uk](mailto:clare.peckitt@rmh.nhs.uk)

Tel: Email:

SM2 5PT

Tel +44 208 915 6634

[nicola.valeri@icr.ac.uk](mailto:nicola.valeri@icr.ac.uk)

|  | **Royal Marsden Scientist** Dr Anguraj Sadanandam  Centre for Molecular Pathology The Royal Marsden  Downs Road Sutton Surrey  SM2 5PT |  | **Royal Marsden Scientist** Dr David Gonzales  Centre for Molecular Pathology The Royal Marsden  Downs Road Sutton Surrey  SM2 5PT |
| --- | --- | --- | --- |
| Tel:  Email: | Tel +44 208 915 6631  [anguraj.sadanandam@icr.ac.uk](mailto:anguraj.sadanandam@icr.ac.uk) | Tel:  Email: | Tel +44 208 915 6525  [david.gonzales-de-castro@icr.ac.uk](mailto:david.gonzales-de-castro@icr.ac.uk) |
|  | **Royal Marsden Clinician Scientist**  Dr Marco Gerlinger  Institute for Cancer Research 237 Fulham Road  London  SW3 6JB |  | **Royal Marsden Clinician Scientist**  Dr Nick Turner  Institute for Cancer Research 237 Fulham Road  London  SW3 6JB |
| Tel:  Email: | Tel +44 (0) 20 7370 5261  [marco.gerlinger@icr.ac.uk](mailto:marco.gerlinger@icr.ac.uk) | Tel:  Email: | Tel +44 (0) 20 7370 5261  [nicholas.turner@icr.ac.uk](mailto:nicholas.turner@icr.ac.uk) |

**University of Leeds Scientist**

Dr Heike Grabsch

Leeds Institute of Molecular Medicine

St James’ University Hospital

Leeds

Tel:

LS9 7TF

Tel +44 (0)113 343 8626

Email: [h.i.grabsch@leeds.ac.uk](mailto:h.i.grabsch@leeds.ac.uk)

## CONTENTS

[**TRIAL ADMINISTRATION 2**](#_bookmark0)

1. [**BACKGROUND 8**](#_bookmark1)
   1. [SUMMARY OF THE STUDY 8](#_bookmark2)
   2. [SCIENTIFIC JUSTIFICATION OF THE STUDY 8](#_bookmark3)
2. [**OBJECTIVES 10**](#_bookmark4)
3. [**STUDY DESIGN 10**](#_bookmark5)
4. [**TISSUE COLLECTION 11**](#_bookmark6)
5. [**TISSUE PROCESSING 11**](#_bookmark7)
6. [**ANALYSIS – BIOMARKERS TO BE STUDIED 12**](#_bookmark8)
   1. [*HER-2* GENE AMPLIFICATION AND PROTEIN OVER-EXPRESSION 12](#_bookmark9)
   2. [POTENTIAL FUTURE THERAPEUTIC TARGETS: HER-3 AND HER-4 EXPRESSION 13](#_bookmark10)
   3. [POTENTIAL MARKERS OF CHEMOTHERAPY SENSITIVITY: ERCC1 13](#_bookmark11)
   4. [POTENTIAL BIOMARKERS OF RESISTANCE TO ANTI-EGFR THERAPY: *KRAS*, *BRAF* AND *PIK3CA* MUTATIONS](#_bookmark12)

[14](#_bookmark12)

- 1. [SEQUENOM MUTATIONAL ANALYSIS 14](#_bookmark13)
  2. [NEXT GENERATION SEQUENCING 14](#_bookmark14)
  3. [FGFR2 GENE AMPLIFICATION & PROTEIN EXPRESSION 14](#_bookmark15)
  4. [GENETIC POLYMORPHISMS THAT MAY AFFECT CHEMOTHERAPY SENSITIVITY (TO BE ANALYSED IN](#_bookmark16)

[SINGAPORE): ERCC1, ERCC2, XRCC1, TS, TP, DPD, OPRT, GSTP1, GSTT1, P53, CYCLIN D1 15](#_bookmark16)

[6.9 PTEN METHYLATION, EXPRESSION AND MUTATIONS (METHYLATION AND MUTATIONS TO BE ANALYSED IN](#_bookmark17)

[SINGAPORE) 16](#_bookmark17)

[GENOME WIDE RNA SCREENING 16](#_bookmark18)

[THIS ANALYSIS WILL BE UNDERTAKEN TO IDENTIFY AMPLIFIED RNA EXPRESSION THAT MAY BE RELEVANT TO](#_bookmark19) [GASTRIC CANCER TUMOURIGENESIS, PROGNOSIS AND CHEMOTHERAPY SENSITIVITY 16](#_bookmark19)

[6.10 .......................................................................................................................................................................... 16](#_bookmark20)

[6.11 GENE COPY NUMBER ANALYSIS 16](#_bookmark21)

[6.12 .......................................................................................................................................................................... 16](#_bookmark22)

- 1. [TUMOUR: STROMA RATIO AS A MARKER OF RESPONSE TO NEO-ADJUVANT THERAPY (TO BE UNDERTAKEN IN](#_bookmark23)

[LEEDS) 16](#_bookmark23)

- 1. [OTHER POTENTIAL BIOMARKERS OF CHEMOTHERAPY SENSITIVITY 16](#_bookmark24)
  2. [ANALYSES OF POTENTIAL PROGNOSTIC BIOMARKERS WHICH MAY BE RELEVANT TO FUTURE RESEARCH](#_bookmark25) [PROTOCOLS 18](#_bookmark25)
  3. [BIOMARKERS OF TUMOUR ANGIOGENESIS: HIF1Α, VEGFR1-3, VEGFA, C, D, PLGF, FGF2 AND](#_bookmark26) [NEUROPILIN 1 AND 2 (IHC) 20](#_bookmark26)

1. [STATISTICAL ANALYSIS 22](#_bookmark27)
   1. [OUTCOME MEASURES 22](#_bookmark28)
   2. [SAMPLE SIZE 22](#_bookmark29)
   3. [PLANNED ANALYSIS 22](#_bookmark30)
2. [SPONSORSHIP 23](#_bookmark31)
3. [INDEMNITY 24](#_bookmark32)
4. [FINANCE 24](#_bookmark33)
5. [PUBLICATION POLICY 24](#_bookmark34)
6. [REFERENCES 26](#_bookmark35)

# Background

- 1. Summary of the Study

The TransMAGIC study collected and analysed tumour tissue collected from patients that participated in the MAGIC trial of chemotherapy before and after surgery compared to surgery alone for patients with operable cancers of the stomach, lower gullet (oesophagus), or the junction between the stomach and gullet (the oesophagogastric junction or OGJ). The percentage of patients that were alive at 5 years in the chemotherapy group was 13% higher than the percentage of patients that only had surgery to treat their cancer. This established chemotherapy before and after surgery as a new standard for operable stomach, lower gullet and OGJ cancers. However, chemotherapy is associated with side effects, some of which can be serious, rarely even life threatening. We know that not all patients benefit from chemotherapy, but do not currently know how to identify such patients. This study will hopefully help us identify markers in the tumour that identify patients more likely to benefit from chemotherapy and those that are likely to be resistant to chemotherapy, known as predictive biomarkers. Additionally, we hope to identify prognostic biomarkers; markers in the tumour that help us to identify patients likely to relapse early, so that they can potentially be selected either for trials of more aggressive treatment, or to avoid futile surgery in the future.

- 1. Scientific Justification of the Study

Worldwide, gastric and oesophageal cancers cause approximately 1.5 million deaths per year.

The MAGIC trial established peri-operative ECF chemotherapy as a standard treatment for operable gastric cancer in the UK, demonstrating a 13% improvement in 5 year survival in patients receiving chemotherapy as well as surgery. However, only 36% of patients will achieve long term survival following this combined modality therapy. It can reasonably be expected that a proportion of patients have an inherently good prognosis and if identified, could be treated with surgery alone and avoid the potential morbidity associated with chemotherapy. Similarly, a subgroup of patients may have an inherently poor prognosis and will relapse despite optimal combination therapy. A further subgroup of patients will attain the greatest benefit from chemotherapy. All cancer researchers are working towards the goal of a personalised approach to cancer treatment, where characteristics of the tumour can be used to determine what combination of treatment is most suitable for an individual patient. This can only be achieved by the study of large banks of tumour tissue collected from this and other large studies in gastric cancer, matching findings in the pathology laboratory to the patient outcomes reported in the trial. In this study, we aim to characterize the tumours of at least 80% of the patients treated with surgery or surgery plus chemotherapy within the UK MAGIC study and correlate the findings

with patient survival. We hope that this will allow us to identify predictive and prognostic biomarkers that can be used to design future clinical trials based upon a personalised approach.

Additionally, we intend to evaluate the incidence and prognostic effect of current and possible future molecular targets for new drugs. To establish the frequency of these molecular targets in localised oesophagogastric cancer is critical to the development of additional therapeutic strategies in this patient group with a relatively poor prognosis despite current best practice treatment. Whilst tissue will be collected prospectively from current clinical trials, the results will not be available for several years. Therefore the analysis of tissue which has already been collected from a randomised study is invaluable to continued progress in this field and will inform the development of future clinical trials.

# Objectives

- 1. To determine whether previously described putative predictive biomarkers of chemo- sensitivity can predict benefit from peri-operative chemotherapy in patients treated within the MRC MAGIC trial.
  2. To describe the frequency of and determine any prognostic effect of potential future molecular targets in oesophagogastric cancer.

# Study Design

Tissue has already been collected from 417/503 MAGIC trial participants and initial analyses have been conducted as part of the ethics approval granted as an amendment to the MAGIC trial in 2006. We intend to carry out the following:

1. Continue the approved ongoing analyses on collected paraffin embedded samples which have been included in tissue microarrays for immunohistochemical and in situ hybridisation analyses, had DNA extracted for sequencing and mutational analyses and RNA extracted for expression analyses.
2. Protein extraction and storage for future analyses.
3. Send one representative (anonymised) slide from each resection to Dr Heike Grabsch at the University of Leeds, for response evaluation. Response evaluation may also be carried out used scanned images of resection specimens. The relative proportion of tumour in resection specimens correlates with survival in colorectal cancer and correlates with response to preoperative therapy. As patients in the MAGIC trial were not radiologically assessed for response to neo-adjuvant therapy, this will provide a potentially useful surrogate

marker of response.

1. Send anonymised extracted DNA and paraffin embedded blocks from resections to Professor Patrick Tan at the National University of Singapore/Duke NUS Graduate Medical School Singapore for analyses. These will include testing for genetic polymorphisms of genes involved in chemotherapy metabolism, testing for changes (including methylation and loss of heterozygosity) in target genes of interest, looking for amplification (increased expression) of genes that may be key to gastric cancer growth including HER2, the target for the drug Trastuzumab (Herceptin) and RNA expression profiling looking for new drug targets.

The results of the translational study will be correlated with clinical outcome measures from the MAGIC trial, namely pathological response rates, progression-free and overall survival. Germline polymorphisms may be correlated with chemotherapy related toxicity. This will allow us to identify both prognostic markers and indirectly identify predictive biomarkers for benefit from

chemotherapy by comparing the effect of the biomarker in patients receiving surgery alone to the effect in those that additionally received chemotherapy.

# Tissue Collection

A TRICC grant was secured by Dr Andrew Wotherspoon and Dr Geoff Chong in May 2006 (Ref C20023/A7217) to collect and analyse biopsies and resections for MAGIC trial participants, without additional consent. Between the ethics approval in April 2006-August 2010, the PIs and/or pathologists from all sites participating in the MAGIC study were contacted by Monica Verma from the MRC Trials Office & Dr Rachel Wong then Dr Alicia Okines from the Royal Marsden Hospital, to request participation in the TransMAGIC study. Nine sites did not participate (The Royal Surrey, Glasgow Royal Infirmary, Whipps Cross, King George’s, Midhurst, Rynland, Nijmegen, Pernambuco, Wellington). Available paraffin embedded tissue blocks from the diagnostic biopsy and/or resection have been collected from 417/503 patients. Tissue suitable for analysis is available for 402 patients; biopsy only in 65 patients, resection only in 156 patients, and paired samples in 181 patients. Five post treatment biopsies in patients that did not subsequently undergo surgery are classified as resections as they are post- treatment samples.

# Tissue Processing

All resections and biopsies have been reviewed by Dr Andrew Wotherspoon and all specimens containing gastric adenocarcinoma have been typed according to Lauren’s classification.

- 1. Selection of areas of tumour for TMA construction: Where multiple tumour-containing blocks were available, a block containing maximum tumour was selected for the TMA construction. Up to 3 areas of tumour were marked on the corresponding H&E slide. Where mixed tumours with heterogenous tumour areas were observed, at least one representative core was selected from each area. Cores for the TMAs are 1mm diameter and 5mm depth.
  2. Selection of blocks for DNA extraction: Resections for which more than one tumour containing block was available, were selected for DNA extraction. Slides were marked for macrodissection where this could be used to increase the tumour concentration. Tumour content was estimated at <30%, 30-50%, >50-70% or >70%. Ten 10µm sections were taken from each selected block.
  3. Selection of paired normal tissue for germline DNA extraction: Where available, the resection doughnut was selected as normal tissue. Five 10µm sections were taken from each selected block.
  4. Selection of blocks for RNA extraction: Blocks containing at least 50% tumour were selected for RNA extraction. After sections had been taken for DNA extraction, a further five 10µm sections were taken from each selected block for RNA.
  5. Blocks from which sections had been taken for RNA extraction will be re-checked for tumour content on an H&E stained section. If sufficient residual tumour is confirmed, these blocks will be sent to Singapore for further RNA extraction for genome wide expression analyses.
  6. Protein may also be extracted from resections for storage for future analyses.

# Analysis – Biomarkers to be Studied

Potential biomarkers are described in the planned order of analysis, based upon an agreed hierarchy.

- 1. *HER-2* gene amplification and protein over-expression

This will be the first analysis undertaken to determine the incidence and effect on prognosis, plus any predictive effect on benefit from ECF chemotherapy. The randomised phase III ToGA study evaluating the addition of Trastuzumab to fluoropyrimidine plus cisplatin chemotherapy for advanced gastric cancer demonstrated a statistically significant benefit from addition of the anti- HER-2 antibody in patients with HER-2 over-expression and/or gene amplification.1 This confirms that HER-2 is an important therapeutic target in gastric cancer. Trastuzumab has now been licensed in the European Union in patients with advanced gastric cancer which is HER-2 positive defined by immunohistochemistry (IHC) 3+ HER-2 over-expression or IHC 2+ over- expression with gene amplification confirmed by in situ hybridisation (ISH). Trastuzumab has also been recommended by the National Institute for Clinical Excellence for HER-2 IHC 3+ patients in combination with cisplatin/fluoropyrimidine chemotherapy.

Previous studies have suggested that HER-2 over-expression is predictive of poor prognosis2 3 4 5 6 7, but this has not been consistently reported.8 An amendment to the MRC ST03 trial of peri- operative chemotherapy with or without bevacizumab for operable oesophagogastric cancer, is currently being prepared to include baseline HER-2 testing and stratified randomisation of HER-

2 positive patients to ECX with or without lapatinib (although funding has yet to be established). The amendment has been designed based upon the expected rate of HER-2 positivity reported in the ToGA trial (approximately 20%),1 although only patients with IHC3+ or IHC2+/ISH+ will be eligible for the lapatinib comparison. However, more recent data suggest that HER-2 over- expression is observed in 7% of primary distal gastric cancers, 24% of oesophagogastric junction tumours9 and 15% of oesophageal adenocarcinomas.10 Quantification of the frequency of HER-2 over-expression and/or gene amplification plus the effect on prognosis and chemo- sensitivity in localised oesophagogastric cancer will allow the amendment to be accurately designed for this population.

- 1. Potential future therapeutic targets: HER-3 and HER-4 expression

HER-3 expression has been found frequently in gastric adenocarcinoma (58-100%)11,12 and is associated with worse T-staging, N-staging, higher rate of distant metastases and worse survival.11 A study in breast cancer has reported a correlation between normal EGFR, HER-2 and HER-3 (but not HER-4) expression and enhanced benefit from anthracycline chemotherapy.13 Relatively little is known about the fourth member of the human epidermal growth factor receptor family. HER-4 over-expression has been reported in 22% of resected gastric tumours evaluated, with no independent prognostic effect reported. However, co- expression of EGFR and HER-4 correlated with poor prognosis.11 In breast cancer, the prognostic effect depends upon the location of the HER-4 expression, which appears to reflect the specific isoform of the receptor that is expressed: Nuclear expression is associated with poorer prognosis and expression of the cleavable isoform, a fragment of which translocates to the nucleus and promotes tumour growth in vitro.14 Specific targeting of this cleavable isoform may therefore be a potential future therapeutic target.

- 1. Potential markers of chemotherapy sensitivity: ERCC1:

The absence of expression of ERCC1, a key protein in nuclear excision repair following DNA damage, is predictive of benefit from cisplatin-based adjuvant chemotherapy in non-small cell lung cancer.15 This biomarker is currently undergoing prospective validation in this setting. Despite numerous studies in oesophagogastric cancer, a significant correlation with outcome has not been consistently demonstrated, which may reflect the sample sizes evaluated and the variable methodology utilised. There are conflicting data regarding the value of the 8F1 antibody clone,16 which was used in the pivotal lung cancer study.15 Evaluation of ERCC1 using both the FL297 and 8F1 clones, with confirmatory evaluation using mRNA expression in a sample of

resections with adequate tumour content, will determine whether ERCC1 is a predictive biomarker for ECF in localised oesophagogastric cancer.

- 1. Potential biomarkers of resistance to anti-EGFR therapy: *KRAS*, *BRAF* and *PIK3CA*

mutations

The presence of *KRAS* mutations, detected in approximately 40% of cases, has been demonstrated to predict for a lack of response to EGFR inhibitors in metastatic colorectal cancer17 18. In oesophagogastric cancer, the incidence of these mutations has not been defined in a large series but is expected to be lower, with recent reports of mutations detected in codons 12 and 13 in up to 13.3%.19 EGFR inhibitors are currently undergoing phase III testing in advanced oesophagogastric cancer, including the Royal Marsden Hospital’s REAL3 study of EOX with or without panitumumab. The presence of a *KRAS* mutation may additionally predict for poor prognosis in gastric cancer.20 In chemotherapy-resistant mCRC, mutations in *BRAF* or *PIK3CA* exon 20 correlated with resistance to cetuximab, with reduced response rate, progression-free and overall survival. In contrast, mutations in exon 9 of *PIK3CA* had no effect on outcome.21 *BRAF* mutations appear to be rare in gastric cancers,22 23 although data are limited. A single study has reported a higher frequency of 11% (2/19 patients) in oesophageal adenocarcinoma,24 but confirmatory data in a much larger series are needed. *PIK3CA* mutations have been reported in 42/264 (16%) gastric adenocarcinomas (81% of which were exon 20 mutations)25 and 3/50 (6%) oesophageal adenocarcinomas (all exon 9).26

- 1. Sequenom mutational analysis:

The Sequenom MassARRAY system will also be used to test for somatic mutations, using Oncocarta panels. Mutations detected by Oncocarta 1.0 include those in *EGFR*, *HER-2*, *MET*, *KRAS* and *PIK3CA*.

- 1. Next generation sequencing

Next generation sequencing of DNA extracted from resections may be conducted in patients with lower oesophageal, junctional and gastric cancers with adequate DNA available.

- 1. *FGFR2* gene amplification & protein expression

Gene amplification and missense mutation in fibroblast growth factor receptor 2 have been described in gastric cancer27,28 and appear to be a late event in carcinogenesis29. *FGFR2* amplification has been reported in approximately 5%30,31 of gastric cancers and is most common in cancers of poorly differentiated, diffuse type histology.31 Over-expression of the FGFR2 protein is similarly most common in diffuse gastric cancers, reported in approximately 30%32,33 of

all gastric cancers and 50% of diffuse cases,34 with inferior survival in patients with over- expression reported.33 FGFR2 targeted agents are currently under evaluation in advanced gastric cancer and the frequency of *FGFR2* amplification is not well described in localised oesphago-gastric cancer.

- 1. Genetic polymorphisms that may affect chemotherapy sensitivity (to be analysed in Singapore): ERCC1, ERCC2, XRCC1, TS, TP, DPD, OPRT, GSTP1, GSTT1, p53, Cyclin D1

Polymorphisms in genes involved in the metabolism of fluoropyrimidines and repairing cisplatin- induced DNA damage have been extensively studied in advanced gastric cancer, but with sparse data from randomised trials to determine whether the effects are truly predictive rather than prognostic. Genes which may be assessed include but are not limited to those listed above. One moderate-sized randomised study of patients with advanced gastric cancer treated with 5-FU, leucovorin and oxaliplatin or 5-FU, leucovorin and cisplatin has reported that the TS- 3R/+6 haplotype, the GSTT1 deletion polymorphism, and genotypes of OPRT-Gly213A and XRCC1-Arg399G were independent predictors of overall survival (OS). For progression-free survival (PFS) analyses, the TS-3R/+6 haplotype and MTR-A2756G were positive predictors of PFS.35 Due to the lack of a control arm, a predictive effect can not be discerned from this study.

A recent meta-analysis of 17 studies of ERCC1 and 2 polymorphisms in colorectal or gastric cancer reported marked racial differences in the significance of polymorphisms: *ERCC1* 11615C>T, T allele was associated with reduced response, progression-free (PFS) and overall survival (OS) in Asians and ERCC2 13181T>G, G allele was associated with reduced response, PFS and OS in Caucasians.36 The XRCC1 194 CT genotype was reported as a prognostic genotype in gastric cancer patients treated with cisplatin plus docetaxel or paclitaxel.37 A further study has identified p53 codon 72 Arg/Pro, GSTP1 codon 105 Ile/Val, and XRCC1 399 Arg/Gln polymorphisms as predictors of outcome following adjuvant oxaliplatin chemotherapy,38 but the lack of control arm again prevents distinction between prognostic and predictive markers. In support of these data, the presence of at least one Val allele at codon 105 of GSTP1 has been reported to predict outcome to first-line therapy with oxaliplatin and 5-FU.39

The A/G; Ile543Val polymorphism in the gene encoding dihydropyrimidine dehydrogenase (DPD) was associated with better prognosis than wild-type in 50 patients with resected gastric cancer treated with adjuvant tegafur.40

Homozygotes for the G allele of codon 241 of cyclin D1 had a poorer prognosis than heterozygotes or AA homozygotes treated with platinum plus 5-FU for advanced gastric cancer.41

6.9 PTEN methylation, expression and mutations (methylation and mutations to be analysed in

Singapore)

Loss of the tumour suppressor gene PTEN appears to be a common event in gastric cancer, reported in 41.5% in a series of 94 samples, where PTEN loss was significantly associated with advanced disease (52.5% vs 2l.2% in early gastric cancer, p=0.003).42 PTEN loss in gastric cancer is commonly due to epigenetic silencing via aberrant promoter methylation.43 Other important mechanisms may include PTEN gene mutations, reported in 28% in a study of 60 advanced gastric cancer, and loss of heterozygosity, reported in 17% of 76 gastric cancers.44 Loss of this tumour suppression gene may be an important event in gastric carcinogenesis, possibly driving tumourigenesis in a subset of patients.

- 1. Genome wide RNA screening This analysis will be undertaken to identify amplified RNA expression that may be relevant to gastric cancer tumourigenesis, prognosis and chemotherapy sensitivity.
  2. Gene copy number analysis

Copy number analyses will be undertaken for *FGFR2, MEK, KRAS* and *EGFR* and other genes on DNA extracted from resections. The rationale for quantifying *FGFR2* amplification and expression has already been described. As the protein expression for each target mayalso be analysed immunohistochemically, the rationale is described for each gene below.

- 1. Tumour: stroma ratio as a marker of response to neo-adjuvant therapy (to be undertaken in Leeds)

The relative proportion of tumour in resection specimens correlates with survival in treatment naive colorectal cancer45 and correlates with response to neo-adjuvant therapy.46 As patients in the MAGIC trial were not radiologically assessed for response to neo-adjuvant therapy, this will provide a potentially useful surrogate marker of response.

- 1. Other potential biomarkers of chemotherapy sensitivity
     1. ***Topo2α* amplification, deletion and expression:** Topoisomerase 2 is the molecular target for anthracyclines such as epirubicin. Amplification and over-expression of the alpha isoform has been associated with benefit from anthracycline-based chemotherapy in breast cancer, but this has not been consistently reported.47 Despite over a decade of research in breast cancer, Topo2α amplification has not been fully validated as a biomarker and at present has no role in routine clinical practice. Co-amplification with *HER-2* is common, possibly related to the location of both genes on chromosome 17. Recent data have suggested that chromosome 17 centromere enumeration probe (CEP

17) duplication may be a more important determinant of anthracycline sensitivity than either HER2 or Topo2α amplification,48 but this remains unresolved.

- - 1. **XPF: Xeroderma pigmentosa group F:** Also known as ERCC4, the protein product of the XPF gene forms a stable complex with ERCC1, which is essential for nucleotide excision repair (NER). The role of the XPF-ERCC1 endonuclease is to initiate NER by incising the DNA, 5’ to the area selected for repair.49 XPF nuclear immunostaining has been reported in 90% of patients undergoing neo-adjuvant therapy in one study, with no correlation with response.50 However, based on a report that commonly used ERCC1 antibodies do not stain specifically for ERCC1,16 a new UK clinical study will allocate patients to different neo-adjuvant chemotherapy regimes based upon XPF staining. Therefore, urgent re-evaluation of this putative predictive biomarker is required.
    2. **TS:** Thymidylate synthetase (TS) is involved in the methylation of the 5-FU metabolite dUMP to dTMP and elevated tumour levels have been correlated with resistance to 5- FU-based chemotherapy.51 However, this association has not been consistently demonstrated.52
    3. **TP:** Thymidine phosphorylase (TP) increases the conversion of 5-FU to its active metabolites. An association between TP expression and poor prognosis has previously been demonstrated in an analysis of 158 gastrectomy specimens53. However, a more recent study of 153 gastrectomy specimens in patients that received adjuvant cisplatin and fluoropyrimidine chemotherapy failed to demonstrate a significant association between TP protein expression and disease-free or overall survival.52
    4. **DPD:** Dihydropyrimidine dehydrogenase (DPD) is the rate-limiting enzyme in 5-FU metabolism. Tumours with high levels of DPD expression may be resistant to 5-FU54 and have been correlated with poor survival.55,56 However, this has not consistently been reported57 and confirmation of DPD as a predictive biomarker is warranted.
    5. **OPRT (Orotate phosphoribosyltransferase):** is an enzyme which activates 5-FU, metabolising it to the active metabolite 5-fluorouridine 5'-monophosphate (FUMP). High OPRT expression would therefore be expected to increase 5-FU availability and response to 5-FU chemotherapy. Increased sensitivity to 5-FU in gastric cancer cell lines over-expressing OPRT has been demonstrated in pre-clinical studies,58 with improved overall survival reported in patients with high OPRT (HR 0.33 (95% CI 0.13 to 0.86) in a retrospective analysis of patients receiving the oral fluoropyrimidine S-1, with or without cisplatin, for advanced gastric cancer. Whether OPRT is a predictive rather than a prognostic biomarker could not be confirmed,59 but could potentially be determined in TransMAGIC.
  1. Analyses of potential prognostic biomarkers which may be relevant to future research protocols

Biomarkers of potential interest which may be analysed in future include but are not limited to:

- - 1. ***EGFR* amplification and expression:** EGFR over-expression has been reported in 44% of gastric resections in a small prospective study,60 and appears to be an independent predictor of poor prognosis.60,61 Similarly, EGFR over-expression is seen in approximately one third 62 to a half 63 of operable oesophageal adenocarcinomas and also correlates with poor prognosis.62 Amplification of *EGFR* in oesophagogastric cancer appears to be less common than in mCRC, reported in just 2.3% of gastric resections64 and 8% of oesophageal cancer resections65 in small case series. Antibodies targeting EGFR are currently undergoing phase III testing in advanced oesophago-gastric cancer.
    2. **IGFR expression:** Expression of IGFR appears to correlate with poor prognosis66 and is therefore a potential therapeutic target in oesophago-gastric cancers, with several IGF1R inhibitors currently undergoing clinical trial evaluation in other solid tumours. IGF1R expression has been reported in 77% of resected gastric cancers and is an independent predictor of poor prognosis.67 Inhibition of IGF1R in gastric cancer xenografts enhances both chemo-and radio-sensitivity and inhibits tumour growth.68
    3. ***MET* gene amplification and protein expression:** Genomic amplification of *MET*, with over-expression of c-met, the receptor tyrosine kinase for hepatocyte growth factor (HGF), has been reported in 10% of gastric cancers, although the overall rate of c-met expression is much higher (46%). Over-expression of c-met has been correlated with poor prognosis69 and is more frequent in patients with liver metastases.70 Gene amplification also correlates with poor prognosis3 and the effects of the associated constitutive receptor activation can potentially be abrogated with selective antibodies or small molecule inhibitors that are currently undergoing clinical evaluation. In oesophageal adenocarcinoma, a similar rate of *MET* amplification has been described (8%).71
    4. ***KRAS* gene amplification and protein expression:** *KRAS* amplification has been reported in 4% 72,73 of primary gastric cancers in small studies and may be the driver gene in such amplified cases, resulting in constitutive MAPK activation in gastric cancer cell lines. 73 Expression of KRAS, detected immunohistochemically, is seen in *KRAS* amplified cell lines.73 The prognostic effect of *KRAS* amplification and expression has not yet been determined.
    5. **ALK (Anaplastic lymphoma kinase):** A receptor tyrosine kinase related to the insulin receptor, constitutively activated ALK due to translocation and formation of a fusion protein was first described in anaplastic lymphoma, but has since been described in several solid tumours including non-small cell lung cancer and oesophageal squamous cell cancers.74 In non-small cell lung cancer, a study of the ALK inhibitor crizotinib, which also inhibits c-met, reported a 57% response rate in patients selected for ALK rearrangements.75 A significant association with signet-ring histology has been reported in lung cancer.76
    6. **mTOR (mammalian target of rapamycin):** A key molecule in intracellular signaling pathways, mTOR is the target of new small molecule inhibitors such as everolimus (RAD001), which is currently undergoing clinical evaluation in advanced gastric cancer. Immunohistochemical detection of phosphor-mTOR in a study of gastric cancer resections correlated with poor disease-free and overall survival, .although statistical significance was lost in the multivariate analysis.77
    7. **Bcl-2:** Bcl-2 suppresses apoptosis, but over-expression may actually suppress proliferation. Higher response rates to chemotherapy have been reported in patients without bcl-2 expression,78 and inhibition of bcl-2 in gastric cancer cell lines enhances cisplatin sensitivity.79 Expression of bcl-2 appears to be more common in gastric cancers with lymph node involvement than in those without.80
    8. **P53:** Mutations in p53, a tumour suppressor gene, are common in gastric cancer, with a trend towards a higher frequency apparent in advanced than early disease.81 Correlation with p53 expression is not reliable.82 However, expression of p53 correlates with poor prognosis in both diffuse and intestinal types of gastric cancer,83 and predicts relapse in resected stage III-IV84 disease. Correlation between p53 expression and relative chemo-resistance has been reported,78 but data are conflicting.50
    9. **P27 (Kip1):** P27 is a G1-S phase checkpoint inhibitor, which maintains cells in the resting state (G0). P27 expression is commonly reduced or lost in gastric cancer and is an independent predictor of reduced survival.85-88 A single pre-clinical report has correlated increased p27 expression with cisplatin resistance in a gastric cancer cell line,89 but clinical data supporting a predictive role for p27 are lacking.
    10. **Mismatch repair (MMR) protein expression (MLH1, MSH2, MSH6, PMS2):** Gastric cancer can occur as part of the Lynch syndrome, usually associated with somatic mutations in the MLH1 or MSH2 genes, leading to microsatellite instability (MSI). MSI due to loss of expression of MLH1 or MSH1 via hypermethylation of the promoter region has been described in series of sporadic gastric cancers with a frequency of up to 15%.90,91,92 There appears to be an association between MSI and good prognosis in stage II gastric cancer.93
    11. **Cyclin D1:** Cyclin D1 is a key protein in cell cycle regulation. Cyclin D1 is not detectable in normal tissues, but has been reported in over one third of localised gastric cancers.94,95 There are contradictory reports of the effect of cyclin D1 expression on prognosis,94,95 and association with intestinal96 or poorly differentiated signet-ring97 histological types. A preclinical report has demonstrated growth suppression and increased chemosensitivity following cyclin D1 inhibition in gastric cancer cell lines.98
    12. **MYC:** C-myc is a well-characterised oncogene, which is frequently amplified in gastric adenocarcinoma (15.5% in one series)99, with reports of protein over-expression in 23.5% in one large series (n=213).100 No significant correlation between c-myc over-expression and prognosis has been consistently demonstrated in gastric cancer.101,102,103
    13. **Survivin:** The anti-apoptosis protein, survivin, has been reported as a prognostic biomarker in gastric cancer, however there are contradictory data supporting both a positive and negative impact on prognosis. A high pre-treatment level has been associated with improved prognosis in a small study of patients that received neo-adjuvant chemotherapy, where it was an independent predictor of survival on multivariate analysis104. However, in a larger study of gastrectomy specimens from patients that had not received neo-adjuvant therapy, tumours positive for survivin expression were associated with a significantly poorer 5 year survival compared to those that were survivin negative 105. It is possible that the survivin levels in the smaller study were affected by neo-adjuvant chemotherapy, as the post treatment levels had no prognostic value. This can potentially be resolved by analysis of survivin in the TransMAGIC study.
    14. **COX2:** COX2 is an inducible isoform of cyclooxygenase, which produces prostaglandin (PG) H2. PGH2 can then be converted to PGE2, which is implicated in both inflammation and tumourigenesis.106 Tumour COX-2 expression is not predictive of response to platinum-based

chemotherapy in colorectal cancer107 and there are conflicting results associating COX-2 expression with prognosis in gastric cancer. 20 84 108

Following review and discussion the TransMAGIC Trial Management Group (TMG) may select other newly identified prognostic or predictive markers for analysis by suitable investigators using next generation sequencing, gene expression profiling or other methods including but not limited to SNP arrays, epigenetic/methylation profiles or microRNA signatures.

- 1. Biomarkers of tumour angiogenesis: HIF1α, VEGFR1-3, VEGFA, C, D, PlGF, FGF2 and neuropilin 1 and 2 (IHC)

These may be prognostic, but there are limited data to suggest a predictive role for response to bevacizumab.

- - 1. **HIF1α (Hypoxia inducible factor-alpha):** HIF1α mediates VEGF-A transcription under hypoxic conditions. A pre-clinical study has reported that HIF1 α contributes to hypoxia- related chemoresistance in gastric cancer.109 Supporting clinical data are currently lacking.
    2. **VEGFR-1:** VEGFR-1 is thought to be less mitogenic than VEGFR-2, possibly acting as a decoy receptor to regulate VEGF-A. Expression in stromal vessels as been reported as an independent predictor of reduced survival in a study of 86 patients that underwent gastric resection.110
    3. **VEGFR-2:** VEGFR-2 is thought to be the principal receptor through which VEGF-A stimulates tumour angiogenesis. However, no prognostic significance of VEGFR-2 expression has been described in one study of resected gastric cancer.111 A second study reported expression of VEGFR-2 in stromal vessels as an independent predictor or reduced survival after gastric cancer resection.110
    4. **VEGFR-3:** VEGFR-3 signalling appears to be principally involved in lymphangiogenesis. Correlation with reduced survival has been reported in resected gastric adenocarcinomas.112
    5. **VEGF-A:** Several studies have reported tumour VEGF expression as a marker of poor prognosis in gastric cancer.61,108,113 Tumour expression of the VEGF-A isoform is also a poor prognostic marker.114
    6. **VEGF-C:** A small retrospective study reported tumour VEGF-C expression as a marker of poor prognosis in 51 patients with resected gastric cancer.114 However, expression was not an independent predictor of poor prognosis in a second study.112
    7. **VEGF-D:** Like VEGF-C, VEGF-D binds VEGFR-3 and has a possible role in lymphangiogenesis. Correlation with lymph node metastases and reduced survival has been reported in resected gastric adenocarcinomas.112
    8. **PlGF (Placental growth factor):** PlGF binds VEGFR-1 and may have a role in tumour angiogenesis. In a study of 79 resected gastric cancers, the level of PlGF expression has been positively correlated with increasing tumour stage, depth of invasion, lymph node expression and worse survival.115
    9. **FGF2 (fibroblast growth factor 2):** FGF2 expression has been detected in 54% of resected gastric cancers analysed in one study, with correlation with poorly differentiated histology and depth of invasion reported.116
    10. **Neuropilin 1 and 2 receptors:** The neuropilin receptors are isoform-specific co- receptors for VEGF-A. Neuropilin 1 may promote angiogenesis when co-expressed with

VEGFR-2.117 Neuropilin-2 has been reported to be up-regulated in the endothelial cells of gastric cancers, enhancing the mitogenic effects of VEGF.118

# Statistical Analysis

- 1. Outcome measures

**Primary endpoint:** Overall Survival

**Secondary endpoint:** Progression-free survival

- 1. Sample Size

As this is a retrospective study, the sample size will be determined by the number of samples available. 437 patients underwent surgery and we aim to collect paired samples from 90% of these. 66 patients did not undergo resection, therefore blocks from the diagnostic biopsy only will be available and we aim to collect 90% of these.

Power calculations have assumed that potential markers will be dichotomised, but an attempt will be made to collect marker information in the form of percentage bands rather than binary data, which will improve power. Where possible, results will be reported and analysed on a continuous scale.

- 1. Planned Analysis

### Power for prognostic factors based on 90% sample retrieval:

For the evaluation of independent, binary, prognostic factors, the study provides ~80% power to detect absolute survival differences at 3 years of 10-15%, dependent upon the prevalence of the markers. The prognostic value of individual markers will be assessed within a proportion hazards regression model which will include other clinical factors of known prognostic importance such as stage, grade and tumour site. The power to detect the independent prognostic value of the markers will be affected by any correlation between the clinical factors and the markers. This is expected to be low, and if up to the order of 0.2, will have little effect on power. To account for the multiple markers being assessed, and because useful prognostic variables will need to demonstrate a substantial effect, a p-value of 0.01 will be used.

### Power for prognostic markers based on 50% sample retrieval:

52% of samples have already been obtained, so allowing for some assay failures this is the minimum sample size anticipated. With ~160 deaths in this population, absolute survival differences of 15-20% (corresponding to hazard ratios of 0.58-0.66 and depending on marker

prevalences) at 3 years would be detectable with 80% power and a significance level of 0.01 as above.

### Power for predictive markers based on 90% sample retrieval:

For the evaluation of predictive factors, ie a differential treatment effect according to the presence or absence of a marker, power will again depend on the prevalence of positive markers, and the level of interaction hypothesised. The overall treatment hazard ratio is 0.75. For the evaluation of predictive factors, a significance cut-off of 0.1 has been used as it is more important here to retain power for potentially important interactions. The study will have ~ 90% power to detect interactions where all the benefit to chemotherapy is confined to those with (or without) the marker of interest (ie a hazard ratio close to 1 in one group, and close to 0.5 in the other, assuming 50% prevalence in both groups), and ~80% power to detect interactions where the majority of benefit is confined to those with (or without) the marker of interest, for example treatment hazard ratios of 0.95 in one group vs 0.55 in the other.

A summary of the interaction effects (ratios of hazard ratios) detectable given the 320 events observed currently in the MAGIC trial is shown in table 1 of appendix 1 for a range of marker prevalences. These represent conservative estimates of power based on dichotomised variables; analysis will also use the data collected as percentage bands or, where available, the continuous data form described above.

### Power for predictive markers based on 50% sample retrieval:

The table in appendix 1 also gives the power to detect interaction effects with sample retrieval rates of 85% (table 2), 75% (table 3) and 50% (table 4) which, as noted above, is the minimum we expect to achieve. In this latter case, we would have approximately 70% power to detect interaction effects where all the benefit to chemotherapy is confined to those with (or without) the marker of interest for marker prevalences of between 40% and 60%.

### Estimates of marker prevalence:

For markers such as B-raf, estimates of the prevalence of mutations will be presented with 95% confidence intervals. The confidence intervals around a 10% prevalence will be narrow (+/-5%) even with 50% sample retrieval, narrowing to +/-3% with 90% of data available.

For the purposes of statistical analysis the MAGIC database may be transferred to RMH for use of RMH statisticians with MRC statisticians retaining oversight as per data transfer agreement.

# Sponsorship

The sponsor of the TransMAGIC trial is the Medical Research Council. Please contact Michael Kilpatrick, Head, MRC Centre London, second Floor, Stephenson House, 158-160 North Gower

Street, London, NW1 2ND up until June 12th 2011. From June 13th 2011, the Medical Research Council’s new address will be Aviation House, 125 Kingsway, London, WC2B 6NH.

# Indemnity

The Samples are a research product. The MRC gives no undertaking, warranty or representation that the Samples are of satisfactory quality, fit for any particular purpose, correspond to any description or sample.

The MRC shall indemnify and hold the Institution harmless from and against all claims (including any claim by any third party against the Institution), liabilities, damages and costs (including legal costs on a full indemnity basis), arising from or related to the actions or omissions of the MRC. The Institution shall not be liable for any damages, costs or expenses, whether special, indirect, incidental, consequential (including lost profit and loss of goodwill, opportunity costs, loss of business, damage to reputation, claims by third parties or customers), exemplary or punitive damages, regardless of the form of action, including in contract or tort (including negligence). The Institution shall not be liable for any loss, damage or corruption of data, howsoever caused.

The MRC acknowledges and agrees that any use of the data derived or results generated from the Institution’s Project and/or the Institution’s Confidential Information by the MRC under this Agreement will be at the sole risk and liability of the MRC. The Institution makes no representation or warranty, whether expressed or implied, with respect to the data and results derived from the Project and/or the Institution’s confidential information, including any representation or warranty as to its accuracy, completeness, merchantability or fitness for a particular purpose or non-infringement on third party proprietary rights.

# Finance

TransMAGIC is sponsored by the MRC Clinical Trials Unit in London. Financial support for the trial has been supplied by Cancer Research UK, grant number: C20023/A7217.

# Publication Policy

It is the intent of the Parties that the findings, methods and results derived from the Project shall be jointly published by the Parties, more specifically authorship of the publication will include (but not be limited to) researchers from the Institution, Royal Marsden Hospital, the MRC CTU and will include appropriate recognition of the authors and other contributors to that publication or presentation in accordance with ordinary scientific custom. If the Parties have not agreed upon a joint publication within one year of the conclusion of the Project, each Party (“Publishing Party”) may, subject to compliance with Clause 3 of the TransMAGIC Clinical Sample Transfer

Agreement (Version 1.0), publish or present at any symposia, national, international or regional professional meeting, academic lecture or in any journal, thesis, dissertation, newspaper or otherwise of its own choosing, the findings, methods and results derived from the Project.

# References

1. Bang YJ, Van Cutsem E, Feyereislova A, et al: Trastuzumab in combination with chemotherapy versus chemotherapy alone for treatment of HER2-positive advanced gastric or gastro-oesophageal junction cancer (ToGA): a phase 3, open-label, randomised controlled trial. Lancet 376:687-97, 2010
2. Garcia I, Vizoso F, Martin A, et al: Clinical significance of the epidermal growth factor receptor and HER2 receptor in resectable gastric cancer. Ann Surg Oncol 10:234-41, 2003
3. Nakajima M, Sawada H, Yamada Y, et al: The prognostic significance of amplification and overexpression of c-met and c-erb B-2 in human gastric carcinomas. Cancer 85:1894-902, 1999
4. Mizutani T, Onda M, Tokunaga A, et al: Relationship of C-erbB-2 protein expression and gene amplification to invasion and metastasis in human gastric cancer. Cancer 72:2083-8, 1993
5. Takehana T, Kunitomo K, Kono K, et al: Status of c-erbB-2 in gastric adenocarcinoma: a comparative study of immunohistochemistry, fluorescence in situ hybridization and enzyme-linked immuno-sorbent assay. Int J Cancer 98:833-7, 2002
6. Tanner M, Hollmen M, Junttila TT, et al: Amplification of HER-2 in gastric carcinoma: association with Topoisomerase IIalpha gene amplification, intestinal type, poor prognosis and sensitivity to trastuzumab. Ann Oncol 16:273-8, 2005
7. Uchino S, Tsuda H, Maruyama K, et al: Overexpression of c-erbB-2 protein in gastric cancer. Its correlation with long-term survival of patients. Cancer 72:3179-84, 1993
8. Grabsch H, Sivakumar S, Gray S, et al: HER2 expression in gastric cancer: Rare, heterogeneous and of no prognostic value - conclusions from 924 cases of two independent series. Cell Oncol 32:57-65, 2010
9. Boers JE, Meeuwissen H, Methorst N: HER2 status in gastro-oesophageal adenocarcinomas assessed by two rabbit monoclonal antibodies (SP3 and 4B5) and two in situ hybridization methods (FISH and SISH). Histopathology, 2011
10. Schoppmann SF, Jesch B, Friedrich J, et al: Expression of Her-2 in carcinomas of the esophagus. Am J Surg Pathol 34:1868-73, 2010
11. Hayashi M, Inokuchi M, Takagi Y, et al: High expression of HER3 is associated with a decreased survival in gastric cancer. Clin Cancer Res 14:7843-9, 2008
12. Sanidas EE, Filipe MI, Linehan J, et al: Expression of the c-erbB-3 gene product in gastric cancer. Int J Cancer 54:935-40, 1993
13. Bartlett JM, Munro A, Cameron DA, et al: Type 1 receptor tyrosine kinase profiles identify patients with enhanced benefit from anthracyclines in the BR9601 adjuvant breast cancer chemotherapy trial. J Clin Oncol 26:5027-35, 2008
14. Junttila TT, Sundvall M, Lundin M, et al: Cleavable ErbB4 isoform in estrogen receptor-regulated growth of breast cancer cells. Cancer Res 65:1384-93, 2005
15. Olaussen KA, Dunant A, Fouret P, et al: DNA repair by ERCC1 in non- small-cell lung cancer and cisplatin-based adjuvant chemotherapy. N Engl J Med 355:983-91, 2006
16. Bhagwat NR, Roginskaya VY, Acquafondata MB, et al: Immunodetection of DNA repair endonuclease ERCC1-XPF in human tissue. Cancer Res 69:6831-8, 2009
17. Amado RG, Wolf M, Peeters M, et al: Wild-type KRAS is required for panitumumab efficacy in patients with metastatic colorectal cancer. J Clin Oncol 26:1626-34, 2008
18. Karapetis CS, Khambata-Ford S, Jonker DJ, et al: K-ras mutations and benefit from cetuximab in advanced colorectal cancer. N Engl J Med 359:1757-65, 2008
19. Stella G RLF, Barone C, Falcone A, Di Fabio F, Martoni A, Lamba S, Ceccarelli C, Siena S, Bardelli A, Pinto C: KRAS and BRAF mutational status as response biomarkers to cetuximab combination therapy in advanced gastric cancer patients, ASCO Annual Meeting. Orlando, J Clin Oncol 2009, pp Abstr e15503)
20. Li M, Liu W, Zhu YF, et al: Correlation of COX-2 and K-ras expression to clinical outcome in gastric cancer. Acta Oncol 45:1115-9, 2006
21. De Roock W, Claes B, Bernasconi D, et al: Effects of KRAS, BRAF, NRAS, and PIK3CA mutations on the efficacy of cetuximab plus chemotherapy in chemotherapy-refractory metastatic colorectal cancer: a retrospective consortium analysis. Lancet Oncol 11:753-62, 2010
22. Kim IJ, Park JH, Kang HC, et al: Mutational analysis of BRAF and K-ras in gastric cancers: absence of BRAF mutations in gastric cancers. Hum Genet 114:118-20, 2003
23. Wu M, Semba S, Oue N, et al: BRAF/K-ras mutation, microsatellite instability, and promoter hypermethylation of hMLH1/MGMT in human gastric carcinomas. Gastric Cancer 7:246-53, 2004
24. Sommerer F, Vieth M, Markwarth A, et al: Mutations of BRAF and KRAS2 in the development of Barrett's adenocarcinoma. Oncogene 23:554-8, 2004
25. Barbi S, Cataldo I, De Manzoni G, et al: The analysis of PIK3CA mutations in gastric carcinoma and metanalysis of literature suggest that exon-selectivity is a signature of cancer type. J Exp Clin Cancer Res 29:32, 2010
26. Phillips WA, Russell SE, Ciavarella ML, et al: Mutation analysis of PIK3CA and PIK3CB in esophageal cancer and Barrett's esophagus. Int J Cancer 118:2644-6, 2006
27. Katoh M, Katoh M: FGF signaling network in the gastrointestinal tract (review). Int J Oncol 29:163-8, 2006
28. Jang JH, Shin KH, Park JG: Mutations in fibroblast growth factor receptor 2 and fibroblast growth factor receptor 3 genes associated with human gastric and colorectal cancers. Cancer Res 61:3541-3, 2001
29. Katoh M: Dysregulation of stem cell signaling network due to germline mutation, SNP, Helicobacter pylori infection, epigenetic change and genetic alteration in gastric cancer. Cancer Biol Ther 6:832-9, 2007
30. Mor O, Ranzani GN, Ravia Y, et al: DNA amplification in human gastric carcinomas. Cancer Genet Cytogenet 65:111-4, 1993
31. Tsujimoto H, Sugihara H, Hagiwara A, et al: Amplification of growth factor receptor genes and DNA ploidy pattern in the progression of gastric cancer. Virchows Arch 431:383-9, 1997
32. Matsunobu T, Ishiwata T, Yoshino M, et al: Expression of keratinocyte growth factor receptor correlates with expansive growth and early stage of gastric cancer. Int J Oncol 28:307-14, 2006
33. Toyokawa T, Yashiro M, Hirakawa K: Co-expression of keratinocyte growth factor and K-sam is an independent prognostic factor in gastric carcinoma. Oncol Rep 21:875-80, 2009
34. Hattori Y, Itoh H, Uchino S, et al: Immunohistochemical detection of K- sam protein in stomach cancer. Clin Cancer Res 2:1373-81, 1996
35. Goekkurt E, Al-Batran SE, Hartmann JT, et al: Pharmacogenetic analyses of a phase III trial in metastatic gastroesophageal adenocarcinoma with fluorouracil and leucovorin plus either oxaliplatin or cisplatin: a study of the arbeitsgemeinschaft internistische onkologie. J Clin Oncol 27:2863-73, 2009
36. Yin M, Yan J, Martinez-Balibrea E, et al: ERCC1 and ERCC2/XPD Polymorphisms Predict Clinical Outcomes of Oxaliplatin-based Chemotherapies in Gastric and Colorectal Cancer: A Systemic Review and Meta-analysis. Clin Cancer Res, 2011
37. Shim HJ, Yun JY, Hwang JE, et al: BRCA1 and XRCC1 polymorphisms associated with survival in advanced gastric cancer treated with taxane and cisplatin. Cancer Sci 101:1247-54, 2010
38. Huang ZH, Hua D, Du X: Polymorphisms in p53, GSTP1 and XRCC1 predict relapse and survival of gastric cancer patients treated with oxaliplatin-based adjuvant chemotherapy. Cancer Chemother Pharmacol, 2009
39. Li QF, Yao RY, Liu KW, et al: Genetic polymorphism of GSTP1: prediction of clinical outcome to oxaliplatin/5-FU-based chemotherapy in advanced gastric cancer. J Korean Med Sci 25:846-52, 2010
40. Grau JJ, Caballero M, Monzo M, et al: Dihydropyrimidine dehydrogenases and cytidine-deaminase gene polymorphisms as outcome predictors in resected gastric cancer patients treated with fluoropyrimidine adjuvant chemotherapy. J Surg Oncol 98:130-4, 2008
41. Stocker G, Ott K, Henningsen N, et al: CyclinD1 and interleukin-1 receptor antagonist polymorphisms are associated with prognosis in neoadjuvant-treated gastric carcinoma. Eur J Cancer 45:3326-35, 2009
42. Im SA, Lee KE, Nam E, et al: Potential prognostic significance of p185(HER2) overexpression with loss of PTEN expression in gastric carcinomas. Tumori 91:513-21, 2005
43. Kang YH, Lee HS, Kim WH: Promoter methylation and silencing of PTEN in gastric carcinoma. Lab Invest 82:285-91, 2002
44. Oki E, Tokunaga E, Nakamura T, et al: Genetic mutual relationship between PTEN and p53 in gastric cancer. Cancer Lett 227:33-8, 2005
45. West NP, Dattani M, McShane P, et al: The proportion of tumour cells is an independent predictor for survival in colorectal cancer patients. Br J Cancer 102:1519- 23, 2010
46. N. West HG, D. Treanor, D. Sebag-Montefiore, H. Thorpe, D. Jayne, H. Rutten, H. A. Swellengrebel, I. , D. Nagtegaal PQ: Quantitative assessment of tumor cell density in rectal cancer following three different preoperative therapies compared to surgery alone. , ASCO Annual Meeting. Chicago, J Clin Oncol, 2010, pp Abstr 3651
47. Oakman C, Moretti E, Galardi F, et al: The role of topoisomerase IIalpha and HER-2 in predicting sensitivity to anthracyclines in breast cancer patients. Cancer Treat Rev 35:662-7, 2009
48. Bartlett JM, Munro AF, Dunn JA, et al: Predictive markers of anthracycline benefit: a prospectively planned analysis of the UK National Epirubicin Adjuvant Trial (NEAT/BR9601). Lancet Oncol 11:266-74, 2010
49. Kuraoka I, Kobertz WR, Ariza RR, et al: Repair of an interstrand DNA cross-link initiated by ERCC1-XPF repair/recombination nuclease. J Biol Chem 275:26632-6, 2000
50. Fareed KR, Al-Attar A, Soomro IN, et al: Tumour regression and ERCC1 nuclear protein expression predict clinical outcome in patients with gastro-oesophageal cancer treated with neoadjuvant chemotherapy. Br J Cancer 102:1600-7, 2010
51. Joshi MB, Shirota Y, Danenberg KD, et al: High gene expression of TS1, GSTP1, and ERCC1 are risk factors for survival in patients treated with trimodality therapy for esophageal cancer. Clin Cancer Res 11:2215-21, 2005
52. Kim JS, Kim MA, Kim TM, et al: Biomarker analysis in stage III-IV (M0) gastric cancer patients who received curative surgery followed by adjuvant 5-fluorouracil and cisplatin chemotherapy: epidermal growth factor receptor (EGFR) associated with favourable survival. Br J Cancer 100:732-8, 2009
53. Takebayashi Y, Miyadera K, Akiyama S, et al: Expression of thymidine phosphorylase in human gastric carcinoma. Jpn J Cancer Res 87:288-95, 1996
54. Toriumi F, Kubota T, Saikawa Y, et al: Thymidylate synthetase (TS) genotype and TS/dihydropyrimidine dehydrogenase mRNA level as an indicator in determining chemosensitivity to 5-fluorouracil in advanced gastric carcinoma. Anticancer Res 24:2455-63, 2004
55. Napieralski R, Ott K, Kremer M, et al: Combined GADD45A and thymidine phosphorylase expression levels predict response and survival of neoadjuvant-treated gastric cancer patients. Clin Cancer Res 11:3025-31, 2005
56. Matsubara J, Nishina T, Yamada Y, et al: Impacts of excision repair cross- complementing gene 1 (ERCC1), dihydropyrimidine dehydrogenase, and epidermal growth factor receptor on the outcomes of patients with advanced gastric cancer. Br J Cancer 98:832-9, 2008
57. Langer R, Specht K, Becker K, et al: Association of pretherapeutic expression of chemotherapy-related genes with response to neoadjuvant chemotherapy in Barrett carcinoma. Clin Cancer Res 11:7462-9, 2005
58. Taomoto J, Yoshida K, Wada Y, et al: Overexpression of the orotate phosphoribosyl-transferase gene enhances the effect of 5-fluorouracil on gastric cancer cell lines. Oncology 70:458-64, 2006
59. Koizumi W, Tanabe S, Azuma M, et al: Impacts of fluorouracil-metabolizing enzymes on the outcomes of patients treated with S-1 alone or S-1 plus cisplatin for first-line treatment of advanced gastric cancer. Int J Cancer 126:162-70, 2010
60. Galizia G, Lieto E, Orditura M, et al: Epidermal growth factor receptor (EGFR) expression is associated with a worse prognosis in gastric cancer patients undergoing curative surgery. World J Surg 31:1458-68, 2007
61. Lieto E, Ferraraccio F, Orditura M, et al: Expression of vascular endothelial growth factor (VEGF) and epidermal growth factor receptor (EGFR) is an independent prognostic indicator of worse outcome in gastric cancer patients. Ann Surg Oncol 15:69- 79, 2008
62. Wang KL, Wu TT, Choi IS, et al: Expression of epidermal growth factor receptor in esophageal and esophagogastric junction adenocarcinomas: association with poor outcome. Cancer 109:658-67, 2007
63. Langer R, Von Rahden BH, Nahrig J, et al: Prognostic significance of expression patterns of c-erbB-2, p53, p16INK4A, p27KIP1, cyclin D1 and epidermal growth factor receptor in oesophageal adenocarcinoma: a tissue microarray study. J Clin Pathol 59:631-4, 2006
64. Kim MA, Lee HS, Lee HE, et al: EGFR in gastric carcinomas: prognostic significance of protein overexpression and high gene copy number. Histopathology 52:738-46, 2008
65. Miller CT, Moy JR, Lin L, et al: Gene amplification in esophageal adenocarcinomas and Barrett's with high-grade dysplasia. Clin Cancer Res 9:4819-25, 2003
66. Matsubara J, Yamada Y, Hirashima Y, et al: Impact of insulin-like growth factor type 1 receptor, epidermal growth factor receptor, and HER2 expressions on outcomes of patients with gastric cancer. Clin Cancer Res 14:3022-9, 2008
67. Matsubara J, Yamada Y, Nakajima TE, et al: Clinical significance of insulin- like growth factor type 1 receptor and epidermal growth factor receptor in patients with advanced gastric cancer. Oncology 74:76-83, 2008
68. Min Y, Adachi Y, Yamamoto H, et al: Insulin-like growth factor I receptor blockade enhances chemotherapy and radiation responses and inhibits tumour growth in human gastric cancer xenografts. Gut 54:591-600, 2005
69. Drebber U, Baldus SE, Nolden B, et al: The overexpression of c-met as a prognostic indicator for gastric carcinoma compared to p53 and p21 nuclear accumulation. Oncol Rep 19:1477-83, 2008
70. Amemiya H, Kono K, Itakura J, et al: c-Met expression in gastric cancer with liver metastasis. Oncology 63:286-96, 2002
71. Miller CT, Lin L, Casper AM, et al: Genomic amplification of MET with boundaries within fragile site FRA7G and upregulation of MET pathways in esophageal adenocarcinoma. Oncogene 25:409-18, 2006
72. Sakakura C, Mori T, Sakabe T, et al: Gains, losses, and amplifications of genomic materials in primary gastric cancers analyzed by comparative genomic hybridization. Genes Chromosomes Cancer 24:299-305, 1999
73. Mita H, Toyota M, Aoki F, et al: A novel method, digital genome scanning detects KRAS gene amplification in gastric cancers: involvement of overexpressed wild- type KRAS in downstream signaling and cancer cell growth. BMC Cancer 9:198, 2009
74. Webb TR, Slavish J, George RE, et al: Anaplastic lymphoma kinase: role in cancer pathogenesis and small-molecule inhibitor development for therapy. Expert Rev Anticancer Ther 9:331-56, 2009
75. Kwak EL, Bang YJ, Camidge DR, et al: Anaplastic lymphoma kinase inhibition in non-small-cell lung cancer. N Engl J Med 363:1693-703, 2010
76. Rodig SJ, Mino-Kenudson M, Dacic S, et al: Unique clinicopathologic features characterize ALK-rearranged lung adenocarcinoma in the western population. Clin Cancer Res 15:5216-23, 2009
77. Xu DZ, Geng QR, Tian Y, et al: Activated mammalian target of rapamycin is a potential therapeutic target in gastric cancer. BMC Cancer 10:536, 2010
78. Kikuyama S, Inada T, Shimizu K, et al: p53, bcl-2 and thymidine phosphorylase as predictive markers of chemotherapy in patients with advanced and recurrent gastric cancer. Anticancer Res 21:2149-53, 2001
79. Lee HK, Lee HS, Yang HK, et al: Prognostic significance of Bcl-2 and p53 expression in gastric cancer. Int J Colorectal Dis 18:518-25, 2003
80. Pan W, Ishii H, Ebihara Y, et al: Prognostic use of growth characteristics of early gastric cancer and expression patterns of apoptotic, cell proliferation, and cell adhesion proteins. J Surg Oncol 82:104-10, 2003
81. Tajima Y, Yamazaki K, Makino R, et al: Differences in the histological findings, phenotypic marker expressions and genetic alterations between adenocarcinoma of the gastric cardia and distal stomach. Br J Cancer 96:631-8, 2007
82. Hanazono K, Natsugoe S, Stein HJ, et al: Distribution of p53 mutations in esophageal and gastric carcinomas and the relationship with p53 expression. Oncol Rep 15:821-4, 2006
83. Lee KE, Lee HJ, Kim YH, et al: Prognostic significance of p53, nm23, PCNA and c-erbB-2 in gastric cancer. Jpn J Clin Oncol 33:173-9, 2003
84. Kim YJ, Kim MA, Im SA, et al: Metastasis-associated protein S100A4 and p53 predict relapse in curatively resected stage III and IV (M0) gastric cancer. Cancer Invest 26:152-8, 2008
85. Mori M, Mimori K, Shiraishi T, et al: p27 expression and gastric carcinoma. Nat Med 3:593, 1997
86. Sgambato A, Migaldi M, Leocata P, et al: Loss of p27Kip1 expression is a strong independent prognostic factor of reduced survival in N0 gastric carcinomas. Cancer 89:2247-57, 2000
87. Nitti D, Belluco C, Mammano E, et al: Low level of p27(Kip1) protein expression in gastric adenocarcinoma is associated with disease progression and poor outcome. J Surg Oncol 81:167-75; discussion 175-6, 2002
88. Galizia G, Ferraraccio F, Lieto E, et al: p27 downregulation and metallothionein overexpression in gastric cancer patients are associated with a poor survival rate. J Surg Oncol 93:241-52, 2006
89. Le TV, Seo Y, Ryu CJ, et al: Increased expression of p27 is associated with the cisplatin resistance in gastric cancer cell line YCC-3. Arch Pharm Res 33:1127-32, 2010
90. Seo HM, Chang YS, Joo SH, et al: Clinicopathologic characteristics and outcomes of gastric cancers with the MSI-H phenotype. J Surg Oncol 99:143-7, 2009
91. Ishiguro K, Kawakami K, Maeda K, et al: Microsatellite instability in gastric cancer is closely associated with hMLH1 hypermethylation at the proximal region of the promoter. Int J Mol Med 12:603-8, 2003
92. Fang DC, Wang RQ, Yang SM, et al: Mutation and methylation of hMLH1 in gastric carcinomas with microsatellite instability. World J Gastroenterol 9:655-9, 2003
93. Beghelli S, de Manzoni G, Barbi S, et al: Microsatellite instability in gastric cancer is associated with better prognosis in only stage II cancers. Surgery 139:347-56, 2006
94. Gao P, Zhou GY, Liu Y, et al: Alteration of cyclin D1 in gastric carcinoma and its clinicopathologic significance. World J Gastroenterol 10:2936-9, 2004
95. Kishimoto I, Mitomi H, Ohkura Y, et al: Abnormal expression of p16(INK4a), cyclin D1, cyclin-dependent kinase 4 and retinoblastoma protein in gastric carcinomas. J Surg Oncol 98:60-6, 2008
96. Muller W, Noguchi T, Wirtz HC, et al: Expression of cell-cycle regulatory proteins cyclin D1, cyclin E, and their inhibitor p21 WAF1/CIP1 in gastric cancer. J Pathol 189:186-93, 1999
97. Feakins RM, Nickols CD, Bidd H, et al: Abnormal expression of pRb, p16, and cyclin D1 in gastric adenocarcinoma and its lymph node metastases: relationship with pathological features and survival. Hum Pathol 34:1276-82, 2003
98. Shuai XM, Han GX, Wang GB, et al: Cyclin D1 antisense oligodexoyneucleotides inhibits growth and enhances chemosensitivity in gastric carcinoma cells. World J Gastroenterol 12:1766-9, 2006
99. Hara T, Ooi A, Kobayashi M, et al: Amplification of c-myc, K-sam, and c- met in gastric cancers: detection by fluorescence in situ hybridization. Lab Invest 78:1143-53, 1998
100. Ninomiya I, Yonemura Y, Matsumoto H, et al: Expression of c-myc gene product in gastric carcinoma. Oncology 48:149-53, 1991
101. Kim YJ, Ghu HD, Kim DY, et al: Expression of cellular oncogenes in human gastric carcinoma: c-myc, c-erb B2, and c-Ha-ras. J Surg Oncol 54:167-70, 1993
102. Songun I, van de Velde CJ, Hermans J, et al: Expression of oncoproteins and the amount of eosinophilic and lymphocytic infiltrates can be used as prognostic

factors in gastric cancer. Dutch Gastric Cancer Group (DGCG). Br J Cancer 74:1783-8, 1996

1. Amadori D, Maltoni M, Volpi A, et al: Gene amplification and proliferative kinetics in relation to prognosis of patients with gastric carcinoma. Cancer 79:226-32, 1997
2. Vallbohmer D, Drebber U, Schneider PM, et al: Survivin expression in gastric cancer: Association with histomorphological response to neoadjuvant therapy and prognosis. J Surg Oncol 99:409-13, 2009
3. Song KY, Jung CK, Park WS, et al: Expression of the antiapoptosis gene Survivin predicts poor prognosis of stage III gastric adenocarcinoma. Jpn J Clin Oncol 39:290-6, 2009
4. Menter DG, Schilsky RL, DuBois RN: Cyclooxygenase-2 and cancer treatment: understanding the risk should be worth the reward. Clin Cancer Res 16:1384-90, 2010
5. Braun MS, Richman SD, Quirke P, et al: Predictive biomarkers of chemotherapy efficacy in colorectal cancer: results from the UK MRC FOCUS trial. J Clin Oncol 26:2690-8, 2008
6. Vidal O, Soriano-Izquierdo A, Pera M, et al: Positive VEGF immunostaining independently predicts poor prognosis in curatively resected gastric cancer patients: results of a study assessing a panel of angiogenic markers. J Gastrointest Surg 12:1005- 14, 2008
7. Liu L, Ning X, Sun L, et al: Hypoxia-inducible factor-1 alpha contributes to hypoxia-induced chemoresistance in gastric cancer. Cancer Sci 99:121-8, 2008
8. Hirashima Y, Yamada Y, Matsubara J, et al: Impact of vascular endothelial growth factor receptor 1, 2, and 3 expression on the outcome of patients with gastric cancer. Cancer Sci, 2008
9. Ozdemir F, Akdogan R, Aydin F, et al: The effects of VEGF and VEGFR-2 on survival in patients with gastric cancer. J Exp Clin Cancer Res 25:83-8, 2006
10. Juttner S, Wissmann C, Jons T, et al: Vascular endothelial growth factor-D and its receptor VEGFR-3: two novel independent prognostic markers in gastric adenocarcinoma. J Clin Oncol 24:228-40, 2006
11. Fondevila C, Metges JP, Fuster J, et al: p53 and VEGF expression are independent predictors of tumour recurrence and survival following curative resection of gastric cancer. Br J Cancer 90:206-15, 2004
12. Ding S, Li C, Lin S, et al: Distinct roles of VEGF-A and VEGF-C in tumour metastasis of gastric carcinoma. Oncol Rep 17:369-75, 2007
13. Chen CN, Hsieh FJ, Cheng YM, et al: The significance of placenta growth factor in angiogenesis and clinical outcome of human gastric cancer. Cancer Lett 213:73-82, 2004
14. Miyamoto N, Yamamoto H, Taniguchi H, et al: Differential expression of angiogenesis-related genes in human gastric cancers with and those without high- frequency microsatellite instability. Cancer Lett 254:42-53, 2007
15. Soker S, Takashima S, Miao HQ, et al: Neuropilin-1 is expressed by endothelial and tumor cells as an isoform-specific receptor for vascular endothelial growth factor. Cell 92:735-45, 1998
16. Kim WH, Lee SH, Jung MH, et al: Neuropilin2 expressed in gastric cancer endothelial cells increases the proliferation and migration of endothelial cells in response to VEGF. Exp Cell Res 315:2154-64, 2009
